# Marine-Derived Diterpenes from 2019 to 2024: Structures, Biological Activities, Synthesis and Potential Applications

**DOI:** 10.3390/md23020072

**Published:** 2025-02-07

**Authors:** Lin Zhang, Debao Li, Xuan Chen, Feng Zhao

**Affiliations:** 1 School of Pharmacy, Yantai University, Yantai 264005, China; zhanglin14010630@163.com; 2 Department of Pharmaceutical and Bioengineering, Zibo Vocational Institute, Zibo 255314, China; cxmgdai@163.com; 3 College of Medicine, Ocean University of China, Qingdao 266000, China; lidebao610@163.com; 4 Shandong Provincial Center for Drug Evaluation, Jinan 250000, China

**Keywords:** marine-derived, diterpenes, structures, biological activities, biosynthesis, applications

## Abstract

Marine diterpenes are an important category of secondary metabolites derived from marine sources, found in a variety of marine animals, plants, and fungi. The increasing diversity of diterpene compounds, along with their unique chemical structures and specific biological activities, have attracted widespread attention. These activities include anti-inflammatory, antiviral, antitumor, antibacterial effects, and therapeutic actions against cancer, with many already developed into clinical drugs. Additionally, as marine medicinal resources continue to be exploited over the long term, the natural resources of many marine diterpene compounds are diminishing, and the biosynthesis of key active components has become a hot topic of research. In this review, we summarize diterpene compounds discovered in the ocean over the past five years, reclassify these compounds, and summarize their structures, biological activities, biosynthesis, and potential applications of marine diterpenoids from 2019 to 2024. This review can provide a reference for the basic research and potential applications of marine-derived diterpene compounds.

## 1. Introduction

Diterpenes are widely distributed in marine organisms, including marine fungi, marine animals and marine plants. The backbones of diterpenes are composed of four isoprene units that join head-to-tail. The diterpenes from marine sources are relatively small in straight chain and monocyclic, mainly in bicyclic and tricyclic diterpenes, especially in oxygen-containing derivatives. The complexity and diversity of diterpenoids make them abundant in biological activities. Many marine diterpenes have been developed as clinical drugs for the treatment of many diseases. For example, compounds isolated from deep-sea fungi have shown antiallergic effects on immunoglobulin E (IgE)-mediated rat basophilic leukemia RBL-2H3 cells [1].

Compared to terrestrial organisms, marine organisms are also one of the sources of bioactive natural products. Montuori et al.’s research indicates that approximately 39,500 marine natural products have been discovered [2]. Among marine organisms, diterpenes are an important class of secondary metabolites [3], they are widely present in marine organisms, including seaweeds, sponges, corals, mollusks, starfish, sea urchins, and certain higher plants [4,5]. So far, thousands of diterpenoid compounds have been isolated and identified [6]. Diterpenoids exhibit beneficial effects on a variety of diseases, including antitumor, antibacterial, antiviral, anti-inflammatory, and anti-cardiovascular disease activities [7]. Due to their diverse biological activities, diterpenoid compounds are widely used in pharmaceuticals, health supplements, cosmetics, and food additives [8]. Currently, various diterpenoids have been discovered in marine, show high diversity and content.

**Figure 1:**
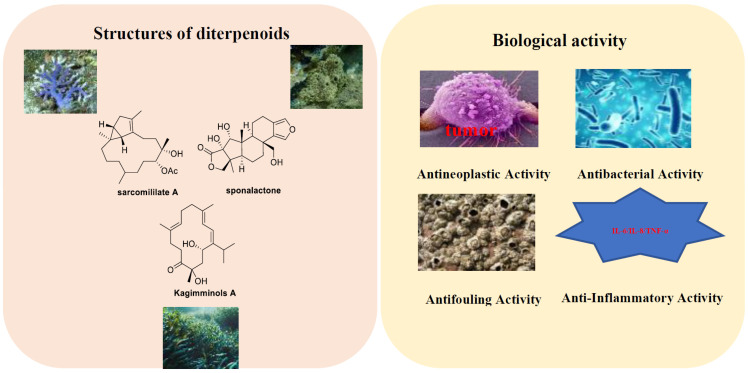
Marine diterpene, including their structures and biological activity.

Diterpenoid compounds are terpenoids containing four isoprene units. Among them, linear and monocyclic diterpenoids are relatively scarce, with bicyclic and tricyclic diterpenoids being the main types, especially those with a high number of oxygenated derivatives. These compounds are widely found in plants, fungi, and marine organisms, exhibiting a variety of biological activities. As important secondary metabolites, diterpenoid compounds derived from marine sources exhibit diverse biological activities, including anti-fouling against marine organisms [9], anti-tumor [10], and antibacterial activities [11]. Marine-derived diterpenoids demonstrate protective effects against various abiotic and biotic stresses, playing a significant role in marine ecosystems. However, the low abundance of marine diterpenoids, combined with the complex growth environments of marine plants, animals, and microorganisms, as well as the intricate extraction and purification processes, fail to meet the demands of research. Therefore, the chemical synthesis of many diterpenoid compounds is also underway. Exploring the biosynthetic pathways of marine-derived diterpenoids for the production of diterpenoids is considered an effective method for the manufacture of these crucial compounds (Figure 1). Figure 1 displays the structures and biological activities of representative marine diterpenes (Figure 1).

In recent years, diterpenoid compounds derived from marine sources have garnered increasing research interest. Therefore, in this review, we systematically summarize the recent research progress on marine-derived diterpenoids in terms of their classification, biological activities, biosynthesis, and potential applications in pharmaceuticals, food, and medicine. Marine-derived diterpenoids have shown potential in the treatment of various diseases, including cancer and inflammatory diseases [12]. New research continuously reveals the novel mechanisms of action and therapeutic potential of these compounds. The value and importance of these compounds in improving global health are becoming increasingly evident, especially in combating drug-resistant pathogens and developing new types of anticancer drugs. The study of marine-derived diterpenes is an interdisciplinary and multi-domain integrated research field. With the advancement of science and technology, our understanding of these compounds will become more profound, thereby better utilizing them to promote human health.

**Figure 2:**
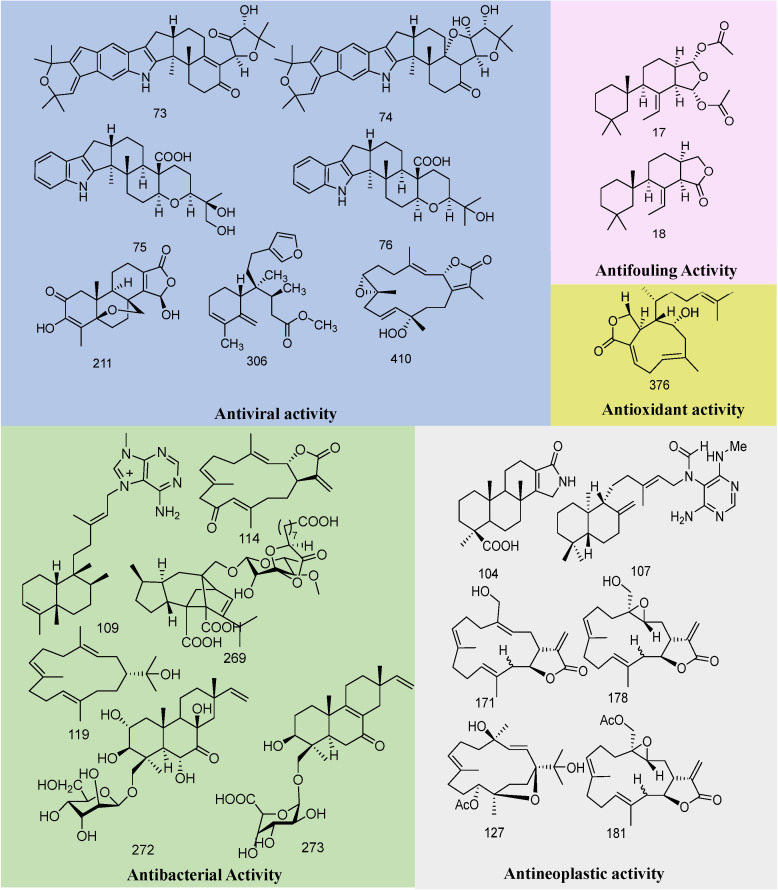

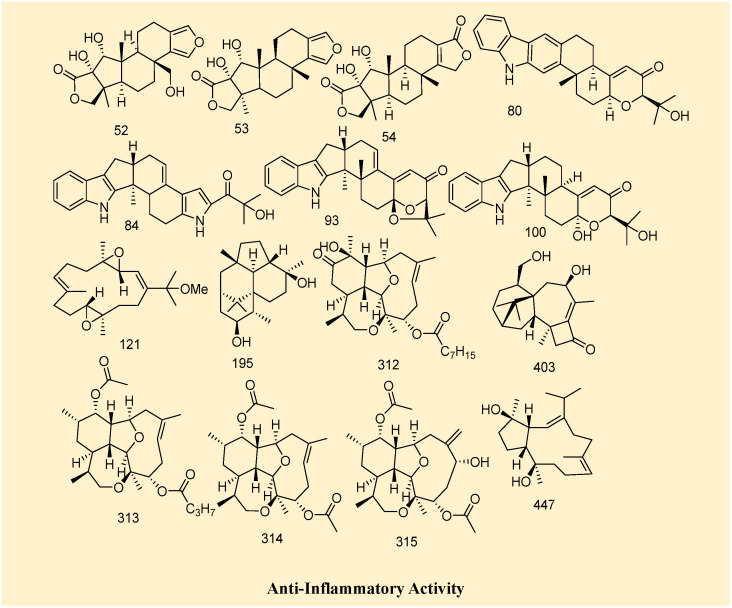
Activity of marine diterpenes (antiviral activity, antibacterial activity, antifouling activity, antioxidant activity, antineoplastic activity, anti-inflammatory activity).

This review first introduces the chemical structures and biological activities of diterpenoid compounds derived from marine sources. It summarizes 414 diterpenoid compounds identified from various marine organisms, including fungi, algae, corals, and sponges, between 2019 and 2024, which exhibit antibacterial, antiviral, antioxidant, and other biological activities. The article provides detailed information on the names, structures, and biological activities of these compounds. Figure 2 displays the biological activities of marine diterpenoids. Furthermore, this article also introduces the synthesis of diterpenoid compounds. It provides an outlook on the application prospects and development trends of marine-derived diterpenoid compounds in the medical field, aiming to offer more references for the research and potential applications of marine diterpenoids.

## 2. Classification of Marine-Derived Diterpenoids

**Figure 3:**
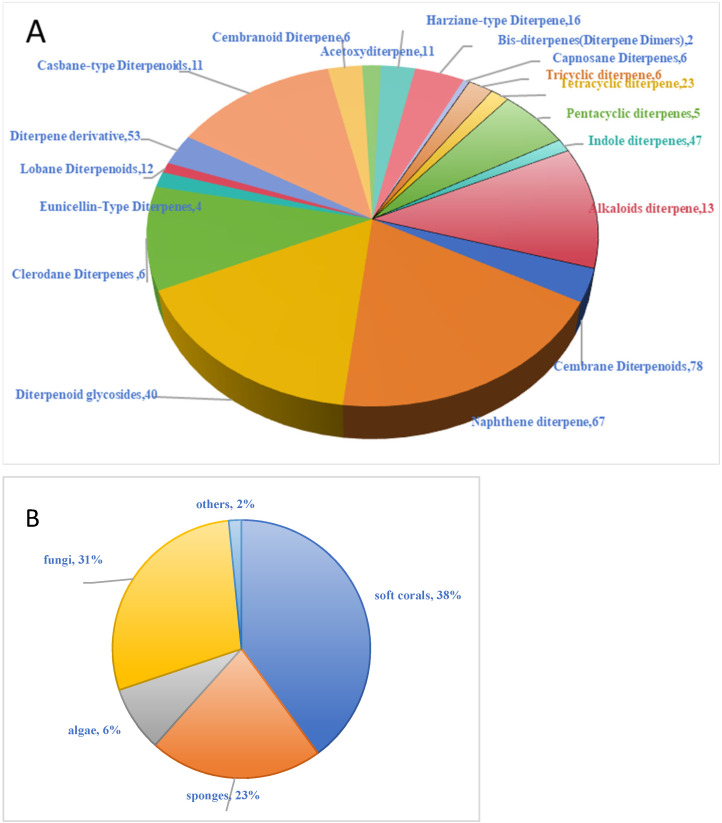
(**A**) Type of the marine-derived diterpenoid (The numbers represent the quantities of the compounds); (**B**) Source of the marine-derived diterpenoids (The numbers represent the percentage of compound sources).

The basic molecular structure of diterpenoid compounds is composed of four isoprene units, which are linked together in a head-to-head or tail-to-tail manner to form different skeletal structures [13]. Diterpenoid compounds can be classified into acyclic (linear), monocyclic, bicyclic, tricyclic, tetracyclic, and some complex diterpenoids based on their chemical structures. Apart from sesquiterpenoids, diterpenoids exhibit the most diverse structural types among terpenoids, which can be generated through cleavage, carbon-carbon bond migration, and skeletal rearrangement. Marine-derived diterpenes are sourced primarily from corals and sponges, with a minority from marine algae. These diterpenes exhibit significantly different structural features compared to those from terrestrial organisms. In this article, we classify marine diterpenes based on their skeletal structures. These classes of diterpenes are widely distributed in marine organisms, possess a variety of biological activities, and are important drug candidates. The frequent skeletal rearrangements in these structures often deviate from the typical head-to-tail arrangement of isoprene units, thus greatly diversifying the terpene structures [14,15]. This review summarizes 447 marine-derived diterpene compounds and their derivatives, including 11 bicyclic diterpenes, 12 tricyclic diterpenes, 28 tetracyclic diterpenes, 5 pentacyclic diterpenes, and 394 other types of diterpene compounds. Figure 3 displays the types and sources of marine-derived diterpenoid.

### 2.1. Acyclic or Monocyclic Diterpenes

Acyclic and monocyclic diterpenes are relatively rare in the ocean. Nonetheless, they still possess specific biological activities. According to new biosynthetic mechanisms, acyclic diterpenes with complex cyclic systems can also be produced [16]. For example, xunnisin is derived from soft corals, while aclatin B acetate and briarein A originate from gorgonian corals.

### 2.2. Bicyclic Diterpenes (***1**–**11***)

In 2020, Vangelis Smyrniotopoulos and colleagues isolated two bicyclic diterpenes, corotrienone (**1**) and an isobrominated dienol (**2**), from the red alga *Sphaerococcus coronopifolius.* The structures of the compounds were determined by extensive spectroscopic analysis [17].

In 2023, Ryosuke Fukada isolated a novel brominated diterpenoid, aplysin-20 aldehyde (**3**), from the Japanese marine red alga *Laurencia venusta Yamada*. The structures of the compounds were determined by extensive spectroscopic analysis, including 1D and 2D Nuclear magnetic resonance (NMR), High-Resolution Atmospheric Pressure Chemical Ionization Mass Spectrometry (HR-APCI-MS), infrared (IR) spectroscopy, and chemical synthesis [18].

**Figure 4:**
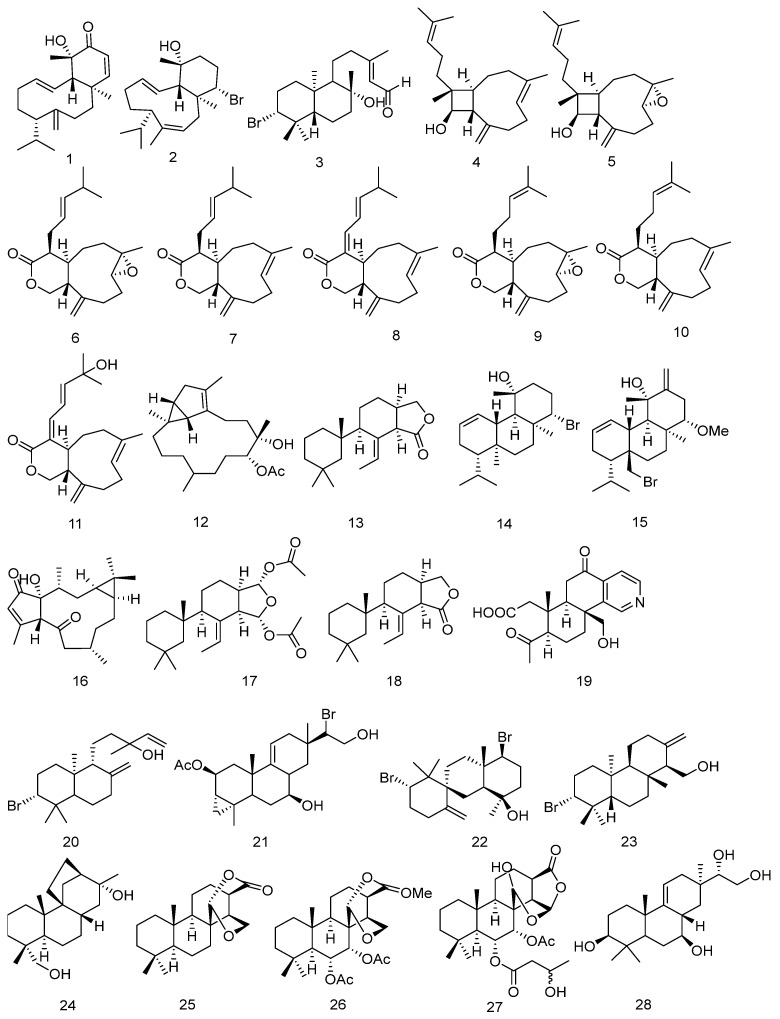

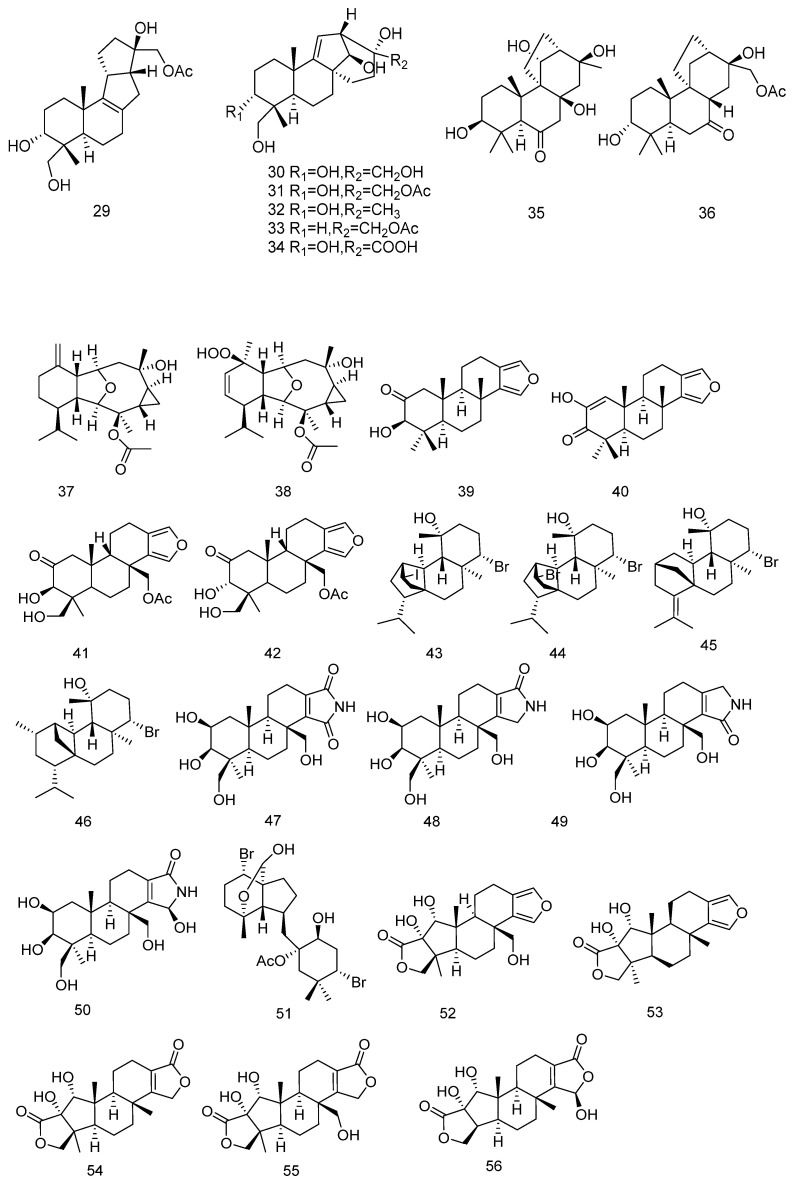
Chemical structures of sesquiterpenoids (**1**–**56**).

In 2024, three novel diterpenoid compounds featuring a rare bicyclic [7.2.0] undec-4-ene skeleton were isolated from the metabolites of the coral *Paragorgia arborea*. These compounds include miolenol, epoxymiolenol, and the xeniolide-class compound epoxycoraxeniolide A. (**4**–**6**). In addition, five known bicyclic diterpenoid compounds **7**–**11** were also obtained. The structure of the new compound was determined through extensive nuclear magnetic resonance (NMR) analysis, and its absolute configuration was established by comparing experimental and time-dependent density functional theory (TDDFT)-calculated electronic circular dichroism (ECD) spectra [19]. The chemical structures of Acyclic or Monocyclic Diterpenes **1**–**11** are depicted in Figure 4.

### 2.3. Tricyclic Diterpene (***12**–**23***)

In 2019, a novel tricyclic hexadecane scaffold diterpenoid compound named sarcomililate A (**12**) was isolated from the Hainanese soft coral *Sarcophyton mililatensis*. The compound possessed a previously undescribed tricyclo[11.3.0.0]hexadecane scaffold. The absolute configuration (AC) of sarcomililate A (**12**) was determined through NMR analysis based on residual dipolar coupling (RDC), ECD calculations, and the Snatzke method [20]. In the same year, Kurnianda Viqqi and colleagues isolated a saturated and polyoxygenated diterpenoid compound, isocopalanol (**13**), containing a three-membered ring, from the acetone extract of the sponge *Callyspongia* sp. The structure of the compound was determined through nuclear magnetic resonance (NMR) [21]. Vangelis Smyrniotopoulos and colleagues isolated two tricyclic diterpenes, with two brominated 6/6/6 tricyclic diterpenes, debromosphaerol (**14**) and the methoxy derivative **15** of 8-hydroxy-dihydro-sphaerococcenol, from the red alga *Sphaerococcus coronopifolius*. The structures of the compounds were determined by extensive spectroscopic analysis [17].

In 2021, a novel diterpenoid compound named sinueretone A (**16**) was isolated from the soft coral *Sinularia erecta* collected in the South China Sea. This compound possesses a novel tricyclic [12.1.0.0] pentadecane carbon framework. The absolute structure was established through extensive spectroscopic analysis, various quantum chemical calculations, and X-ray diffraction analysis [22].

In 2022, two nor-diterpenes, namely 9,11-dihydrogracilin A (**17**) and the novel compound 9,11-dihydrogracillinone A (**18**) were isolated from the sponge *Dendrilla antarctica* [23]. In the same year, Liang Yongqian and their colleagues isolated a novel dinorspongiane-type diterpenoid, dinorspongiapyridine (**19**), with a pyridine ring at the D-ring from the sponge *Spongia* sp. The structures and absolute configurations of these compounds were determined through nuclear magnetic resonance spectroscopy, single-crystal X-ray diffraction and comparison with literature methods [24]. Maria Harizani and her partners isolated two diterpenes, isopinnatol B (**20**) and deoxyparguerol (**21**), from the red algae of the genus *Laurencia*. The structures of the compounds were determined by comparison with known literature [25].

In 2023, Mohamed A. Tammam and her partners isolated a tricyclic diterpene, kahukuene B (**22**), from the red alga *Laurencia majuscula* collected at the Red Sea’s Hurghada reef. The structure of the compound was determined through extensive analysis of NMR and MS data [26]. Ryosuke Fukada isolated a novel brominated 13-dehydroxyisoaplysin-20 (**23**), from the Japanese marine red alga *Laurencia venusta Yamada*. The structures of the compounds were determined by extensive spectroscopic analysis, including 1D and 2D NMR, HR-APCI-MS, IR spectroscopy, and chemical synthesis [18]. The chemical structures of Tricyclic Diterpene are depicted in Figure 4.

### 2.4. Tetracyclic Diterpene (***24**–**51***)

In 2019, a tetracyclic compound named Stemarane diterpene (**24**), featuring a unique hydrocarbon skeleton, was isolated from a species of seaside purslane *Stemodia maritima* L. Its structure was determined through chemical and spectral analysis [27]. In the same year, colleagues isolated three new sponge diterpenes, aplyroseols **25**–**27**, from two specimens of the marine sponge *Dendrilla rosea* collected from the Australian archipelago. The structures and relative configurations of the compounds were determined through spectroscopic analysis (2D NMR) [28]. Siwen Niu and his team isolated a novel cembrane diterpenoid compound Botrytis-cembrane A (**28**) from the deep-sea fungus *Botryotinia fuckeliana* [29]. The absolute configuration of this compound was elucidated through extensive spectral analysis and ECD calculations. At the same time, eight diterpenoid compounds namely Botrycin A–H (**29**–**36**) were also isolated for the first time, they possess novel carbon skeletons with tetracyclic frameworks of 6/6/5/5 (**29**), 6/6/5/6 (**30**–**34**), and 6/6/6/5 (**35**–**36**). The structures of the compounds were determined through extensive spectral analysis, chemical derivatization, and quantum chemical calculations of ECD and OR data [30]. Geng Li and his team isolated two new polyoxygenated diterpenes named klyflaccilides A and B (**37**–**38**) from the Hainan soft coral *Klyxum flaccidum*. They possess a rare 6/5/8/3 tetracyclic system. Their structures were determined through extensive spectral analysis, X-ray diffraction analysis, and quantum chemical calculation methods [31]. Dawrin Pech-Puch and his team isolated two new spongian furanoditerpenes, β-hydroxyspongia-13(16),14-dien-2-one and 19-dehydroxy-spongian diterpene (**39**–**40**) from the sponge *Spongia tubulifera*. The planar structures of the new compounds were determined through 1D/2D NMR and IR spectral analysis, as well as high-resolution electrospray ionization mass spectrometry (HRESIMS). The absolute configurations were determined by ECD spectrum, with calculations performed using time-dependent density functional theory (TDDFT) [32]. Qian Chen and his team isolated two new spongian diterpenes, 17-O-acetylepispongiatriol and 17-O-acetylspongiatriol (**41**–**42**) from the sponge *Spongia officinalis* collected in the South China Sea. The structure of the compound was elucidated through spectroscopic analysis, and its absolute configuration was determined based on ECD analysis [33].

In 2020, Vangelis Smyrniotopoulos and colleagues isolated four tetracyclic halogenated diterpenes, namely iodocoronol (**43**), bromocoronol (**44**), bromotetrasphaereniol (**45**), and the methoxy derivative **46** of ioniol I, from the red alga *Sphaerococcus coronopifolius*. The structures of the compounds were determined by extensive spectroscopic analysis [17].

In 2022, four novel spongian diterpenes D–G (**47**–**50**) were first isolated from the sponge *Spongia* sp. The structures of these compounds were elucidated through spectroscopic analysis and quantum chemical methods, and their absolute configurations were determined [34].

In 2023, Misaki Nagasaka and colleagues isolated a new irieane-type diterpene, 12-hydroxypinnaterpene C (**51**), from the Okinawan sea hare *Aplysia argus*. The structure of the compound was determined by extensive spectroscopic analysis, including NMR and HR-ESI-MS [35]. The chemical structures of Tetracyclic Diterpene are depicted in Figure 4.

### 2.5. Pentacyclic Diterpenes (***52**–**56***)

In 2019, a 5,5,6,6,5-pentacyclic diterpene, Sponalactone (**52**) was first isolated from the sponge *Spongia officinalis* collected from the South China Sea. The structure of the compound was determined through spectroscopic analysis, and its absolute configuration was established based on ECD analysis [33].

In 2021, a rare A-ring contracted diterpenoid, 17-dehydroxysponalactone (**53**) was isolated from the Red Sea sponge *Spongia* sp. The structure and relative and absolute configurations of the compound were determined through Overhauser effect (NOE) correlation analysis and comparative biosynthesis [36].

In 2022, three new 5,5,6,6,5-pentacyclic sponge diterpenes with a β-hydroxy group at the C-1 position, Spongenolactones A–C (**54**–**56**) were isolated for the first time from the Red Sea sponge *Spongia* sp. The structures of these new compounds were determined through extensive spectroscopic analysis, and their absolute configurations were established by comparing the experimental circular dichroism (CD) spectra with the calculated ECD spectra [37]. The chemical structures of Pentacyclic Diterpenes are depicted in Figure 4.

### 2.6. Other Types of Diterpenes

#### 2.6.1. Indole Diterpenes (**57**–**103**)

In 2019, two new compounds, epipaxilline (**57**) and penerpene J (**58**) were isolated from the marine-derived fungus *Penicillium* sp. Their structures, including absolute configurations, were determined based on spectroscopic methods and ECD analysis [38].

In 2021, four new indole diterpenoid compounds named penerpenes K–N (**59**–**62**) were isolated from the marine-derived fungus *Penicillium* sp. Through extensive one-dimensional (1D) and two-dimensional (2D) nuclear magnetic resonance (NMR), high-resolution electrospray ionization mass spectrometry (HRESIMS) data spectroscopic analysis and ECD calculations, the structures of the compounds were determined [39].

In 2022, six new indole diterpene amino acid conjugates, noonindoles A–F (**63**–**68**) were isolated for the first time from the marine-derived fungus *Aspergillus noonimia*. Additionally, four known analogs, paspaline, paspaline B, carboxylic acid, and emindole SB (**69**–**72**) were also isolated. The structures of these compounds were determined through extensive spectroscopic analysis and X-ray crystallography analysis [40].

In 2022, Zhang Yahui and colleagues isolated two unusual indole diterpenoid derivatives, Oxalierpenes A and B (**73**–**74**) from the marine-derived fungus *Penicillium oxalicum*. The absolute configurations of compounds were determined using calculated TDDFT ECD and DP4+ methods. Oxalierpene A (**73**) is the first indole diterpenoid derivative featuring a 4-hydroxy-5,5-dimethyldihydrofuran-3-one pentacyclic side chain. Oxalierpene B (**74**) possesses a unique 6/5/6/5/5/6/6/5/5 ring system [41].

In 2022, Sen Pang and their colleagues isolated a novel indole diterpenoid compound, Penijanthine E (**75**) along with a known analogue (**76**) from the marine-derived fungus *Penicillium citrinum*. The absolute configuration of Penijanthine E (**75**) was determined through calculated TDDFT ECD and DP4+ calculations. Meanwhile, the absolute configuration of compound **76** was confirmed by single-crystal X-ray diffraction analysis and TDDFT ECD calculations [42].

**Figure 5:**
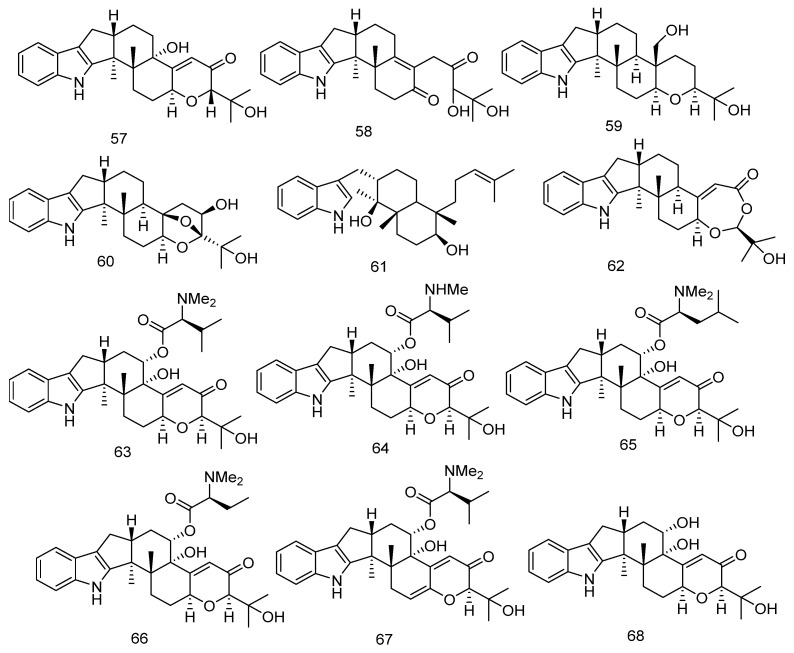

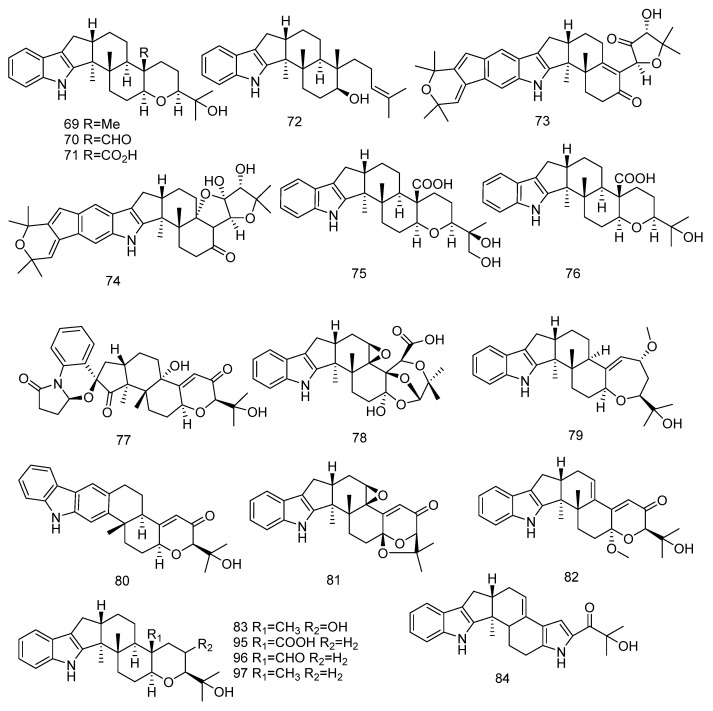

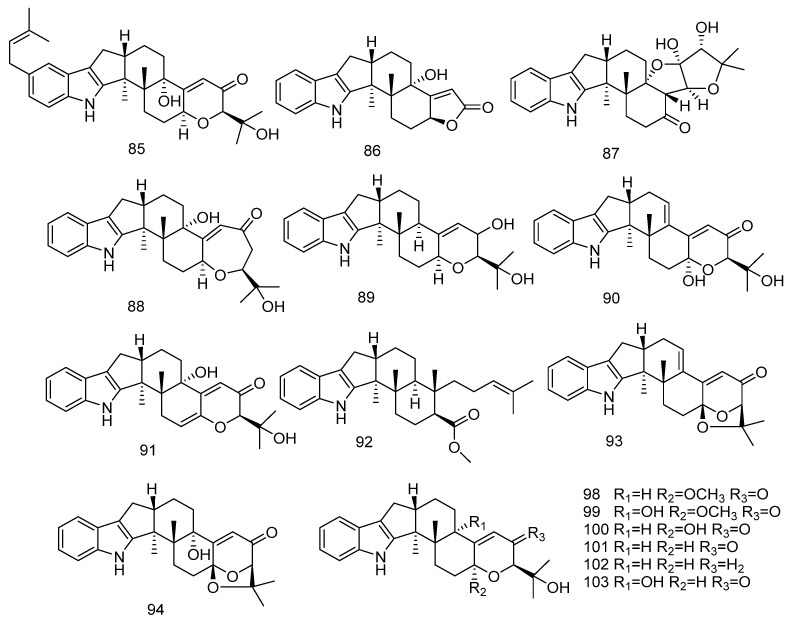
Chemical structures of sesquiterpenoids (**57**–**103**).

In 2024, seven novel indole diterpenoid compounds, namely penpaxilloids A–E (**77**–**81**), 7-methoxypaxilline-13-ene (**82**), and 10-hydroxy-paspaline (**83**) were isolated from the marine-derived fungus *Penicillium* sp. Additionally, 20 known compounds (**84**–**103**) were also isolated. The structures of the novel compounds were elucidated through extensive spectroscopic analysis, combined with NMR calculations analyzed by DP4+ and ECD calculations [43]. The chemical structures of Indole diterpenes are depicted in Figure 5.

#### 2.6.2. Alkaloids Diterpene (**104**–**113**)

In 2019, three novel diterpenoid alkaloids, ceylonamide G–I (**104**–**106**) were isolated from the Indonesian sponge Spongia sp. The chemical structures of these compounds were determined through spectroscopic analysis [44].

In 2020, Changhoon Choi and his colleagues isolated a diterpenoid alkaloid, (−)-agelamide D (**107**) from the sponge *Agelas* sp. [45].

**Figure 6:**
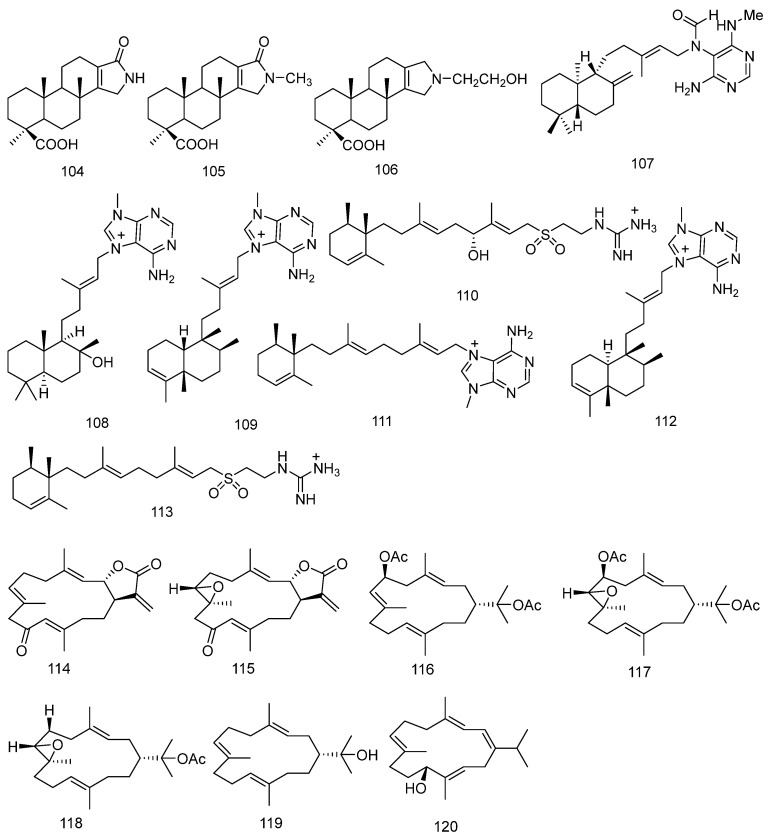

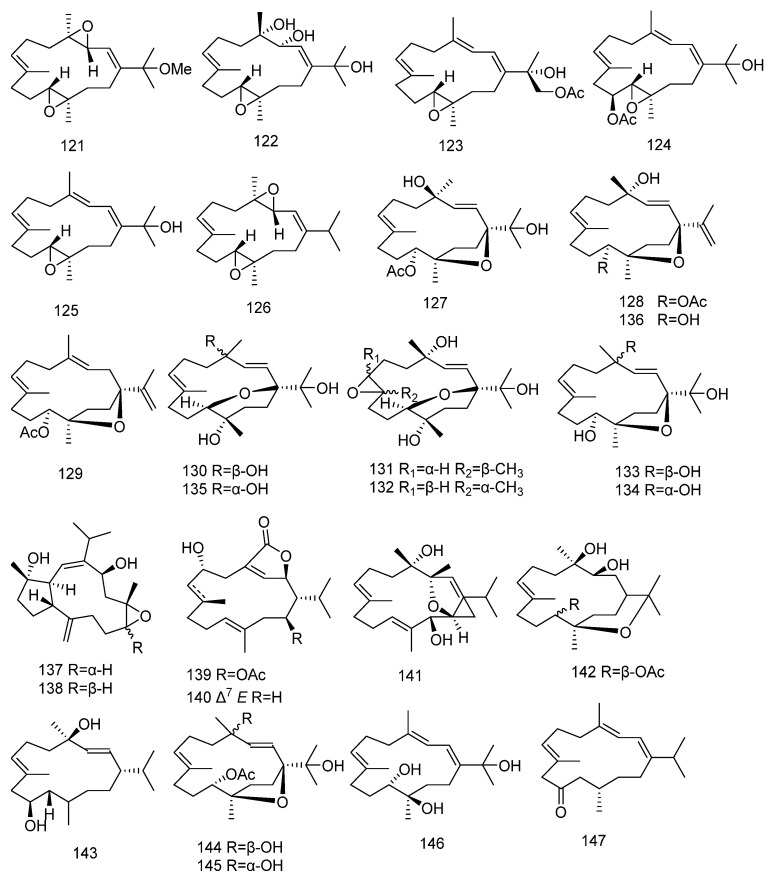

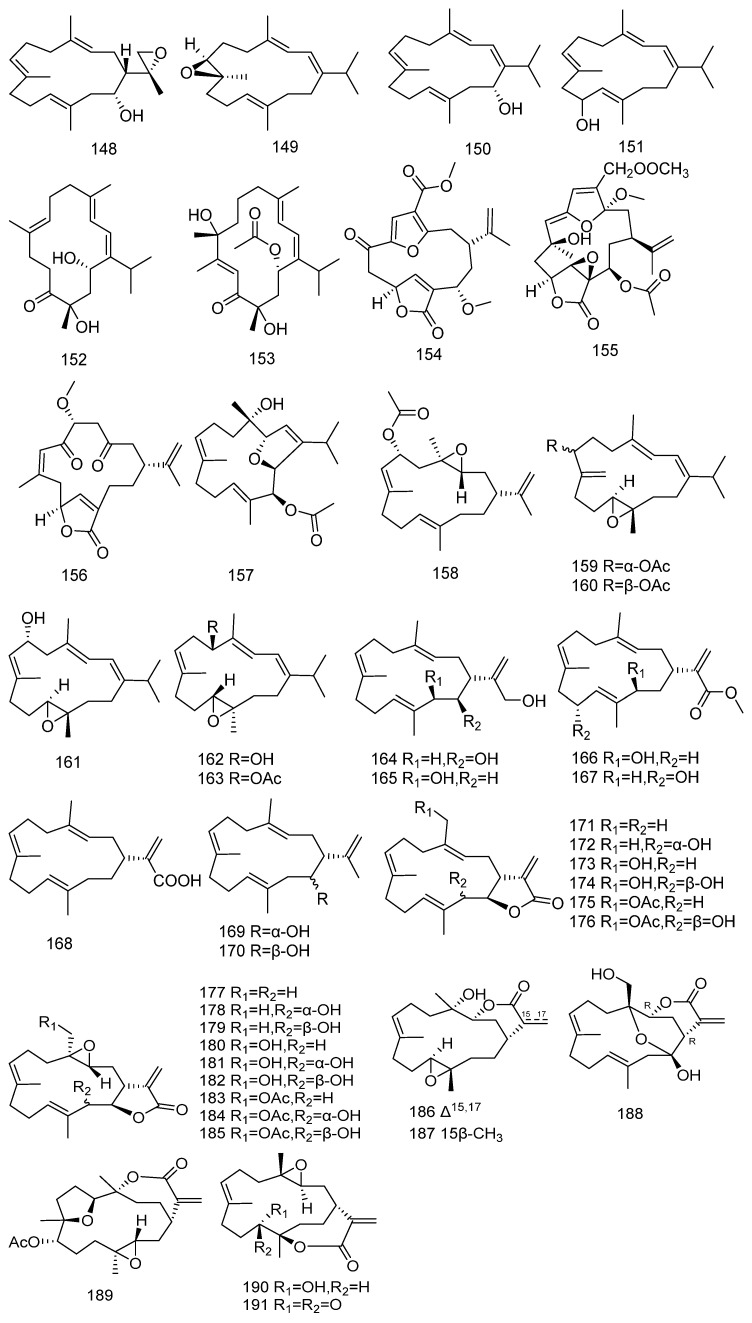
Chemical structures of sesquiterpenoids (**104**–**191**).

In 2022, three new diterpene alkaloids, (+)-8-epiagelasine T, (+)-10-epiagelasine B and (+)-12-hydroxyagelasidine C (**108**–**110**) were isolated from the sponge *Agelas citrina*. Additionally, three known compounds, (+)-ent-agelasine F, (+)-agelasine B, and (+)-agelasidine C (**111**–**113**) were also isolated. The structures of these compounds were determined through extensive 1D and 2D NMR, HRESIMS, and by comparing them with literature data. Notably, (+)-ent-agelasine F was isolated as a natural product for the first time [46]. The chemical structures of Alkaloids Diterpene are depicted in Figure 6.

#### 2.6.3. Cembrane Diterpenoids (**114**–**191**)

Cembrane diterpenoids are characterized by a 14-membered carbon ring and a variety of functional groups, and they are found in both marine and terrestrial organisms. Numerous studies have demonstrated that cembrane diterpenoids possess cytotoxic and anti-inflammatory activities, making them widely used in the development of new drugs.

In 2019, three new cembrane diterpenes, nephthecrassocolides A–B, and 6-acetoxy nephthenol acetate were isolated from one population of *Nephthea* sp. (**114**–**116**). In addition, along with three known compounds, 6-acetoxy-7,8-epoxy nephthenol acetate, epoxy nephthenol acetate, and nephthenol (**117**–**119**). The structures of these compounds were determined through spectroscopic analysis [47].

In 2022, five new flexible cembrane-type macrocyclic diterpenes, sarcomililatols C-G (**120**–**124**) were isolated from the soft coral *Sarcophyton mililatensis*. Additionally, two known analogs **125** and **126** were also isolated. The structures and absolute configurations of the natural macrocyclic compounds **120**–**126** were determined through extensive spectroscopic analysis, X-ray diffraction analysis, time-dependent density functional theory/electronic circular dichroism (TDDFT ECD) calculations, chemical reactions, and the modified Mosher method [48].

In 2023, six new cembrane diterpenes containing non-aromatic oxacycles, namely sarcoxacyclols A–F (**127**–**132**) were isolated from the Hainan soft coral *Sarcophyton mililatensis*. Additionally, four known analogs (**133**–**136**) were also isolated. The structures of the compounds were determined through extensive spectroscopic analysis and comparison with literature data. Furthermore, the absolute configuration of sarcoxacyclols D was established by X-ray diffraction analysis [49]. In the same year, Yuting Song and colleagues discovered six new polyoxygenated diterpenes, sartrocheliols A–E (**137**, **139**, **141**–**144**) from the South China Sea soft coral *Sarcophyton trocheliophorum*. Additionally, four known diterpenoid compounds **138**, **140**, **145**, and **146** were isolated. Through extensive spectroscopic data analysis, sartrocheliol A (**127**) was identified as a rare capnosane-type diterpene, and sartrocheliols B–E (**128**–**131**) as cembrane-type diterpenes. Their absolute configurations were determined by QM-NMR, modified Mosher method, and X-ray diffraction analysis [50]. Shenghui Zhu and colleagues isolated two new oxygenated cembrane-type diterpenes, situmulins A (**147**) and B (**148**) from the soft coral *Sinularia tumulosa*. Additionally, three oxygenated cembrane-type diterpenes **149**–**151** were also isolated. The structures of situmulins A and B (**147**–**148**) were established through extensive 1D and 2D NMR spectral data analysis and comparison with known compounds. The absolute configuration of situmulins A (**147**) was determined by TDDFT combined with ECD methods, while the relative configuration of situmulins B (**148**) was assigned via quantum mechanical-NMR (QM-NMR) calculations [51].

In 2024, two new cembrene diterpenoids, Kagimminols A and B (**152**–**153**) were isolated from the marine cyanobacterium *Okeania* sp. By combining DP4 analysis with an efficient NMR chemical shift calculation protocol, the relative configurations of the compounds were determined without consuming the precious natural products. The absolute configurations of the compounds were established through ECD spectroscopy [52]. Additionally, research has shown that Kagimminols A and B (**152**–**153**) exhibit selective growth inhibitory activity against the pathogen of human African trypanosomiasis. In the same year, Cili Wang and colleagues isolated five new cembranes, sinudenoids H–L (**154**–**158**) from the South China Sea soft coral *Sinularia densa*. The structures and absolute configurations of the compounds were determined through extensive spectroscopic data analysis, single-crystal X-ray diffraction, comparison with literature data, and quantum chemical calculations [53]. Shenghui Zhu and colleagues isolated five new cembrane diterpenes, lobocalines A–E (**159**–**163**) from the Chinese soft coral *Lobophytum catalai* Tixier-Durivault. The structures of the compounds were established through extensive spectroscopic analysis, NMR calculations combined with DP4+ analysis, time-dependent density functional theory-electronic circular dichroism (TDDFT-ECD) calculations, X-ray diffraction analysis and comparison with reported spectroscopic data from the literature [54]. Shoumao Shen and colleagues isolated a series of polyoxygenated cembrane macrocyclic diterpenes (**164**–**191**) from the Hainan soft coral *Lobophytum crassum*. This includes three new flexible cembranoids, lobophycrasins E–G (**165**–**167**), and twenty-five known analogues. The structures of the compounds were determined through extensive spectroscopic data analysis, quantum mechanics-nuclear magnetic resonance (QM-NMR) methods, the modified Mosher method, X-ray diffraction analysis, and comparison with literature-reported data [55]. The chemical structures of Cembrane Diterpenoids are depicted in Figure 6.

#### 2.6.4. Naphthene Diterpene (**192**–**264**)

In 2019, three new cycloalkane-type diterpenes, conidiogenols C–D and conidiogenone L (**192**–**194**) were isolated from the marine-derived fungus *Penicillium* sp. The structures of the compounds were determined through extensive spectroscopic data analysis, and their absolute configurations were further established by ECD calculation analysis [56]. In the same year, Yamada T. and colleagues isolated three new types of diterpenoid compounds, trichodermanins F–H (**195**–**197**) from the fungus *Trichoderma harzianum* OUPS-111D-4 derived from the sponge *Halichondria okadai*. These compounds feature a 6-5-6-6 fused ring system. The structures of the compounds were elucidated by NMR and high-resolution fast atom bombardment mass spectrometry (HRFABMS) spectral analysis, and their absolute configurations were determined by the Mosher method and CD spectroscopy [57].

In 2020, three new rare cycloalkane diterpenes (**198**–**200**) were isolated from the marine sediment-derived fungus *Penicillium* sp. The structures of the compounds were determined through HRESIMS and NMR analysis, and the absolute configurations of the compounds were further established by X-ray crystallography experiments [58]. In the same year, Liang Yongqian and colleagues isolated twelve new norspongian diterpenes, dinorspongians A–F (**201**–**206**) and epoxynorspongians A–F (**207**–**212**) from the sponge *Spongia* sp. Among them, compounds **201**–**206** are the first sponge diterpenoid compounds with a new 3,4-seco-3,19-dinorspongian diterpene skeleton to be isolated. Compounds **207**–**212** are the first 19-norspongian diterpenes containing a 5,17-epoxy unit to be isolated. The absolute configuration structures of these compounds were determined by methods such as NMR, X-ray diffraction, and quantum chemical calculations [59]. Yongqi Tian and colleagues isolated four new homoverrucosane-type diterpenes (**213**–**216**) and two known analogs (**217**–**218**) from the marine sponge *Halichondria* sp. The structures and absolute configurations of the compounds were determined through spectroscopic analysis, theoretical calculations, and comparison with literature data [60].

In 2021, a heptadecylphenol derivative, 1-methyloxy-3-hydroxy-4-methyl-5- heptadecylphenol (**219**) was isolated from the South China Sea sponge *Luffariella variabilis*. The structure of the compound was determined through NMR and MS analysis. The absolute configuration of the compound was established by NOESY experiments, ECD spectroscopy, and DP4+ probability analysis [61]. In the same year, Hye Jin Kim and colleagues isolated a new sesquiterpene, acrepseudoterin (**220**) from the Antarctic lichen-derived fungal strain *Acremonium* sp. The compound inhibits enzyme activity in a dose-dependent manner, with an IC_50_ value of 22.8 ± 1.1 µM, and was identified as a competitive inhibitor of PTP1B [62].

In 2022, five new dolabellane diterpenes, clavularinlides A–E (**221**–**225**) were isolated from the soft coral *Clavularia inflata* collected from the South China Sea. The structures of these compounds were determined through extensive 1D and 2D NMR, high-resolution electrospray ionization mass spectrometry (HRESIMS), calculated ECD, and DP4+ probability analysis [63]. In the same year, Thomas Majer and colleagues isolated two new ircinianin-type compounds, ircinianin lactone B and ircinianin lactone C (**226**–**227**) from the sponge *Ircinia wistarii*. Additionally, five known compounds (**228**–**232**) from the ircinianin compound family were also isolated. Ircinianin lactones B and C are new ircinianin terpenoids with a modified oxidation pattern. The structures of the compounds were determined through extensive high-resolution electrospray ionization mass spectrometry (HRESIMS) and 1D/2D NMR techniques, as well as computational chemistry and quantum mechanical calculations [64]. Qing Bu and his colleagues isolated three diterpenoid compounds, mililatensols A–C (**233**–**235**), with rare sarsolenane and capnosane skeletons for the first time from the soft coral *Sarcophyton mililatensis* collected in the South China Sea. The structures of mililatensols A-C were elucidated through extensive spectroscopic analysis and comparison with literature data. The absolute configuration of compound mililatensols B was determined through TDDFT and ECD calculations [65]. Jinhua Hu and her team isolated three novel phomopsin-type diterpenoids, neocucurbins A–C (**236**–**238**) and their derivatives neocucurbins D–G (**239**–**242**) from the marine-derived fungus *Neocucurbitaria unguis-hominis* for the first time. These compounds share a novel polyoxygenated 5/6/12 or 5/6/13 fused tricyclic system. Neocucurbins D-G possess a 5/6 fused bicyclic system with an opened macrocycle, which is a first in the phomopsin family. The absolute configurations of these compounds were established through spectroscopic data analysis, single-crystal X-ray diffraction experiments, and ECD calculations [66].

In 2023, a novel briarane diterpene, briarlide S (**343**) was isolated from the soft coral *Pachyclavularia violac*. Additionally, six known compounds, namely briarlides A, B, E, G, H (**244**–**248**), and brianodin B (**249**) were also isolated. The structures of these compounds were elucidated through Fourier Transform Infrared Spectroscopy (FTIR), NMR, and HR-ESI-MS analyses. The relative stereochemical structure of briarlide S was determined using Nuclear Overhauser Effect Spectroscopy (NOESY) [67]. In the same year, Anton N. Yurchenko and colleagues isolated two new cycloalkane diterpenes, 4-hydroxyleptosphin C (**250**) and 13-epi-conidiogenone F (**251**) from the marine sediment-derived fungus *Penicillium antarcticum*. The absolute stereostructures of these cycloalkane diterpenes were established through an extensive application of the Mosher method and quantum chemical calculations of ECD spectra [68]. Hsuan-Jung Tseng and colleagues isolated a new diterpenoid, Sinulariaone A (**252**), with a 13-membered carbon ring skeleton from a species of the octocoral *Sinularia*. The structure of the compound was determined by extensive spectroscopic analysis, computational calculations, and X-ray diffraction analysis [69].

**Figure 7:**
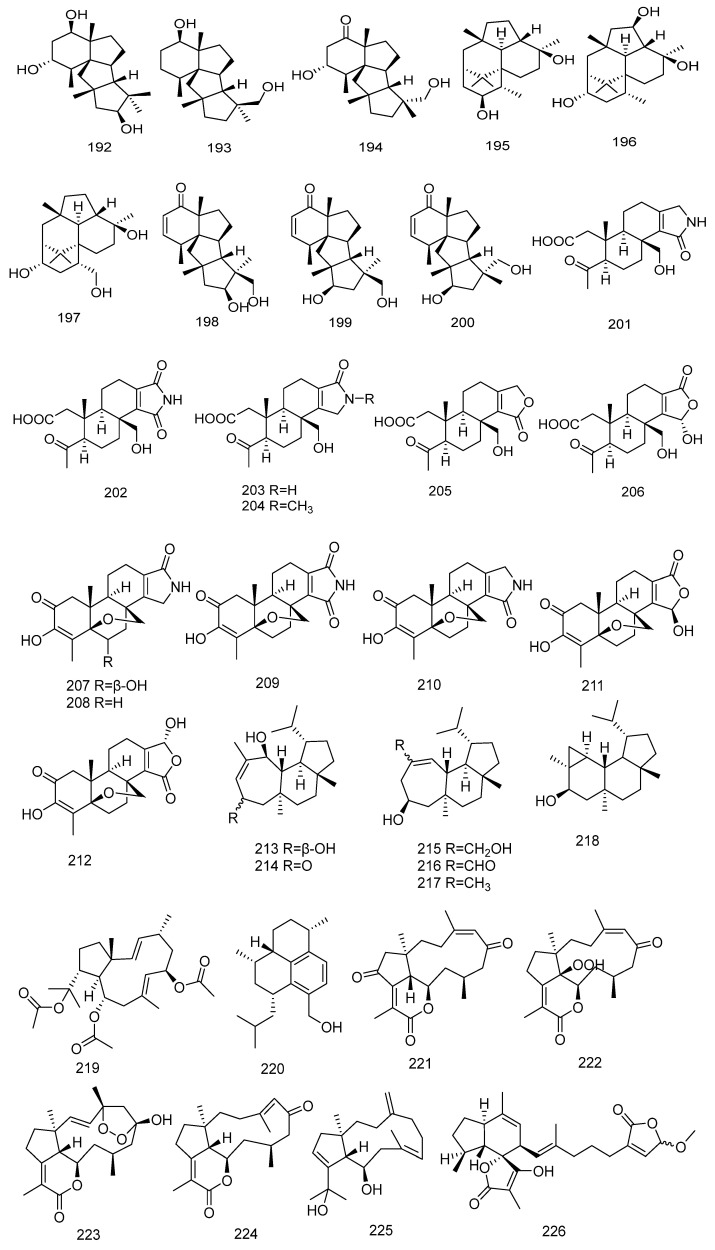

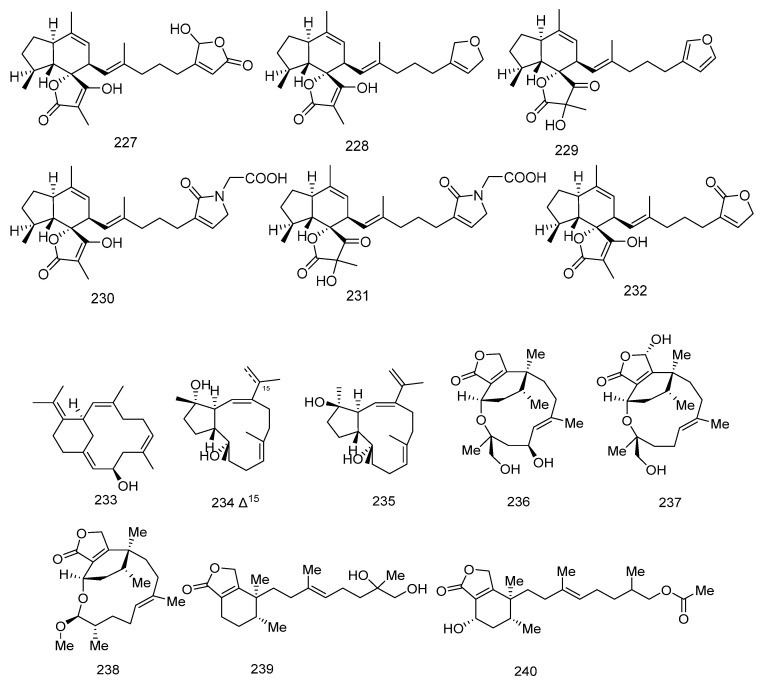

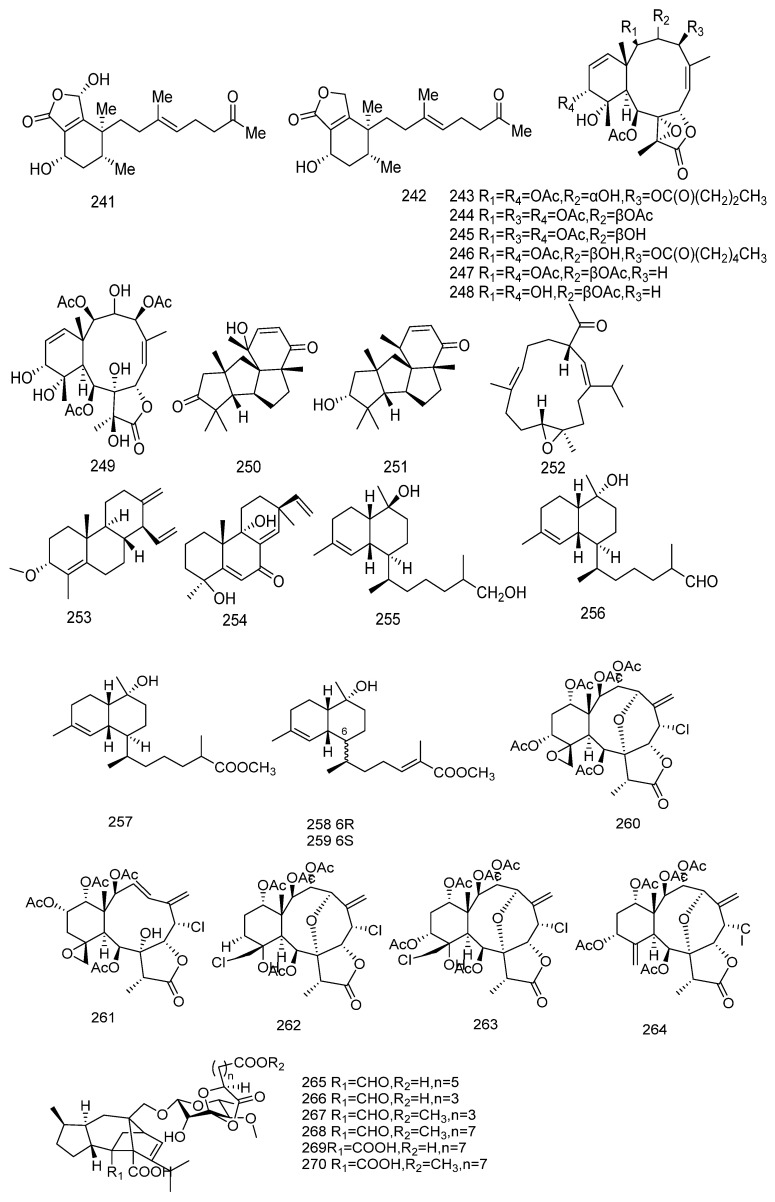

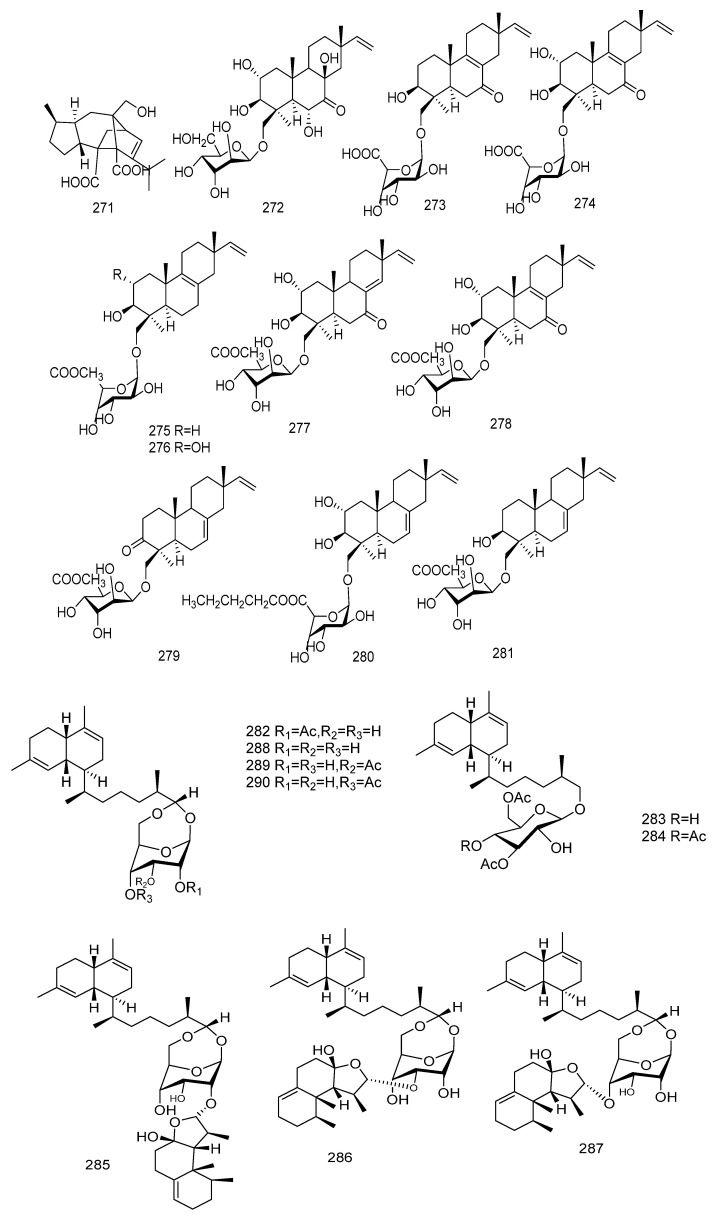

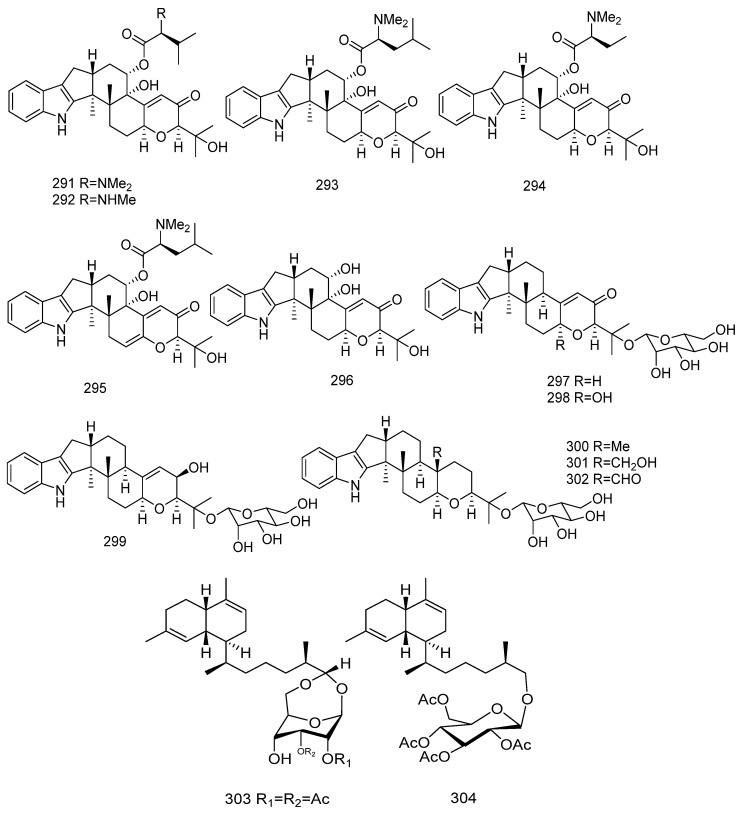
Chemical structures of sesquiterpenoids (**192**–**304**).

In 2024, two 19-nor diterpenoids, talascortene H (**253**) and talascortene I (**254**) were isolated and identified from the sea anemone-derived endophytic fungus *Talaromyces scorteus*. The structures of these compounds were determined through spectral data analysis, single-crystal X-ray diffraction, tandem mass spectrometry, and ECD calculations [70]. In the same year, five new biflorane-type diterpenoids, biofloranates E–I (**255**–**259**) were isolated from the soft coral *Lemnalia bournei* collected in the South China Sea. The chemical structures and stereochemistries of these compounds were determined through extensive spectroscopic methods, TDDFT and ECD calculations, and comparisons with existing literature [71]. Hai Nhat Do and colleagues isolated five chlorinated briarane-type diterpenoids, gemmacolide X (**260**), frajunolide I (**261**), fragilide F (**262**), 12α-acetoxyfragilide F (**263**), and 12α-acetoxyjunceellin (**264**), from the octocoral *Junceella fragilis*. The structures of the compounds were determined by single-crystal X-ray diffraction analysis and 2D NMR spectroscopy [72]. The chemical structures of Naphthene Diterpene are depicted in Figure 7.

#### 2.6.5. Diterpenoid Glycosides (**265**–**304**)

In 2019, a new cyclosporine-type tetracyclic diterpenoid compound, cyclosporine B (**265**) was isolated from the marine fungus *Curvularia hawaiiensis.* Additionally, six novel cyclosporine-type tetracyclic diterpenoid glycosides, moriniafungins B–G (**266**–**271**), containing the rare spiro[1,3-dioxolan-4-one] ring, were isolated for the first time from marine-derived fungi. The structures of these new compounds were determined through comprehensive spectroscopic data analysis and mass spectrometry [73]. In the same year, Olesya I. Zhuravleva and colleagues isolated ten new diterpenoid glycosides, virescenosides Z9–Z18 (**272**–**281**) from the marine-derived fungus *Acremonium striatisporum*. The structures of the isolated compounds were established based on spectroscopic methods [74].

In 2021, six new bicyclic diterpenoid glycosides were isolated from the soft coral *Lemnalia bournei*, including three novel compounds, lemnaboursides E-G (**282**–**284**), and three additional novel compounds, lemnadiolboursides A–C (**285**–**287**). Additionally, three known lemnaboursides (**288**–**290**) were also isolated. The structures of these compounds were determined through extensive spectroscopic analysis, ECD analysis, chemical methods and comparisons with literature data [75].

In 2023, Sarani Kankanamge and colleagues isolated six indole diterpenes (IDTs), noonindoles A–F (**291**–**296**) from the marine sand-derived fungus *Aspergillus noonimiae* in Western Australia. Chemical characteristic analysis was used to detect and prioritize novel compounds, while cultivation characteristic analysis was employed to optimize production conditions, leading to the isolation of the first IDT glycosides, noonindoles G-L (**297**–**302**). The structures of these compounds were determined through extensive spectroscopic analysis and biosynthetic considerations [76].

In 2024, two new bicyclic diterpenoid glycosides, lemnaboursides H–I (**303**–**304**) were isolated from the soft coral *Lemnalia bournei* collected in the South China Sea. The chemical structures and stereochemistries of these compounds were determined through extensive spectroscopic methods, TDDFT and ECD calculations, and comparisons with existing literature [71]. The chemical structures of Diterpenoid Glycosides are depicted in Figure 7.

#### 2.6.6. Clerodane Diterpenes (**305**–**311**)

Clerodane diterpenoids represent a large class of naturally occurring bicyclic diterpenes that have garnered increasing attention in recent years due to their broad and potent biological activities [77].

In 2019, six clerodan-type diterpenoids, namely raspailol, raspadiene, kerlinic acid, its methyl ester, annonene, and 6-hydroxyannonene (**305**–**310**) were isolated from the sponge *Raspailia bouryesnaultae*. Among them, raspadiene is a novel diterpene with a skeletal rearrangement that was isolated for the first time. The structure of this compound was determined through HRMS and NMR data analysis [78].

**Figure 8:**
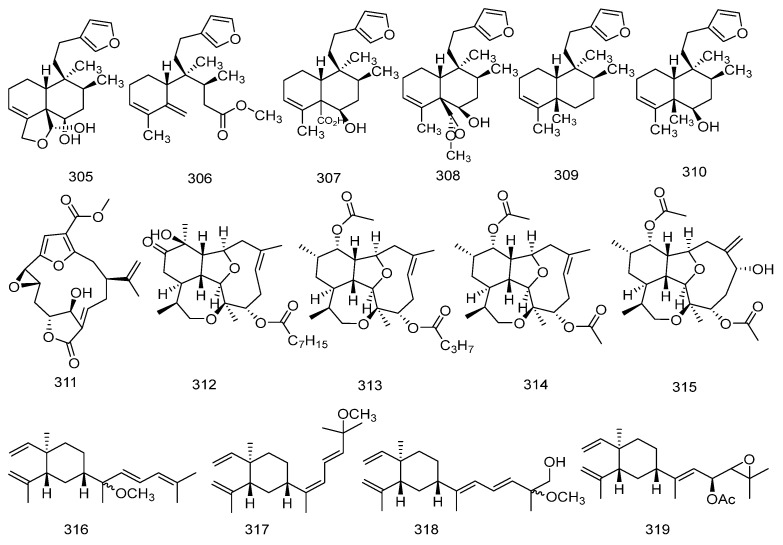

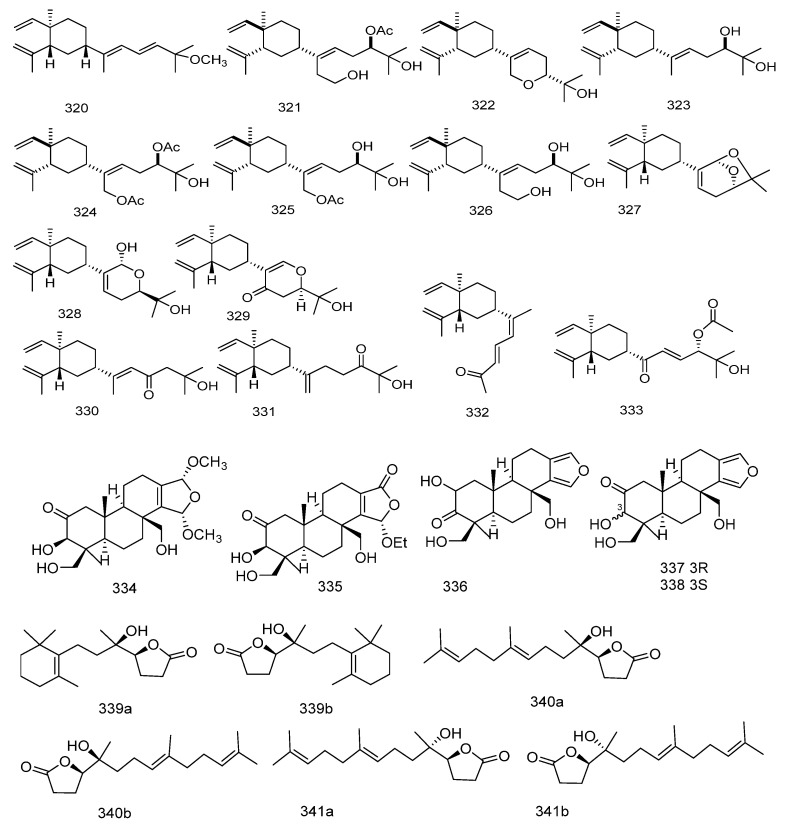

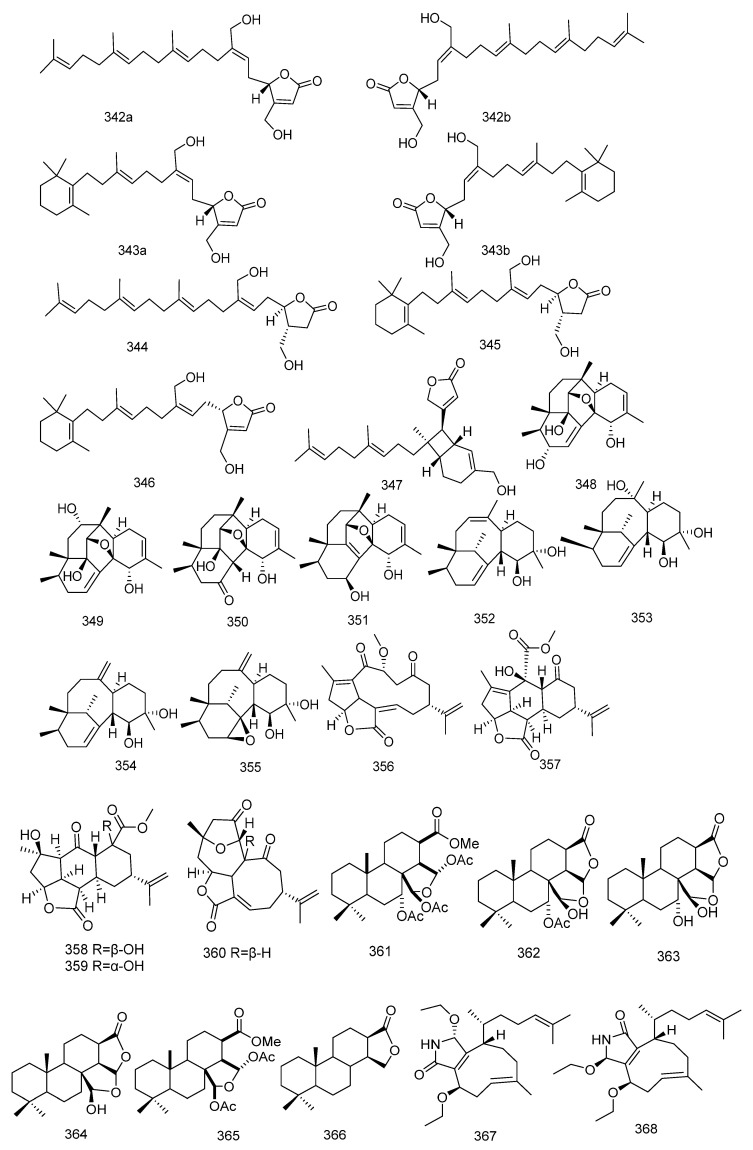

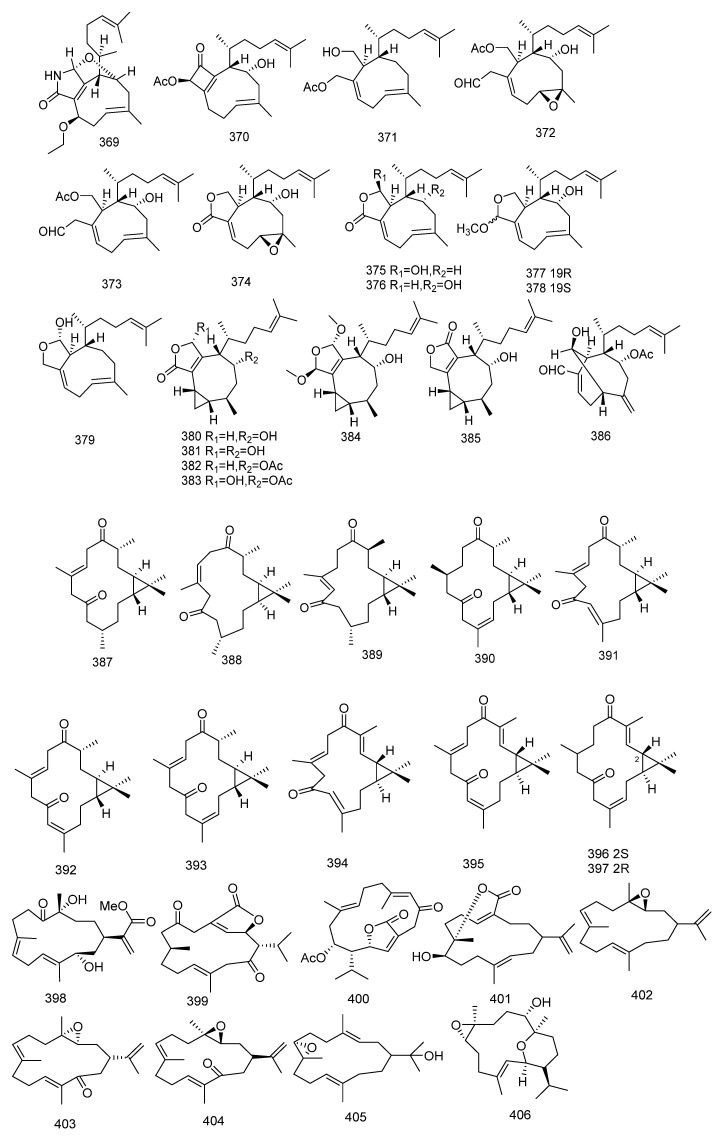

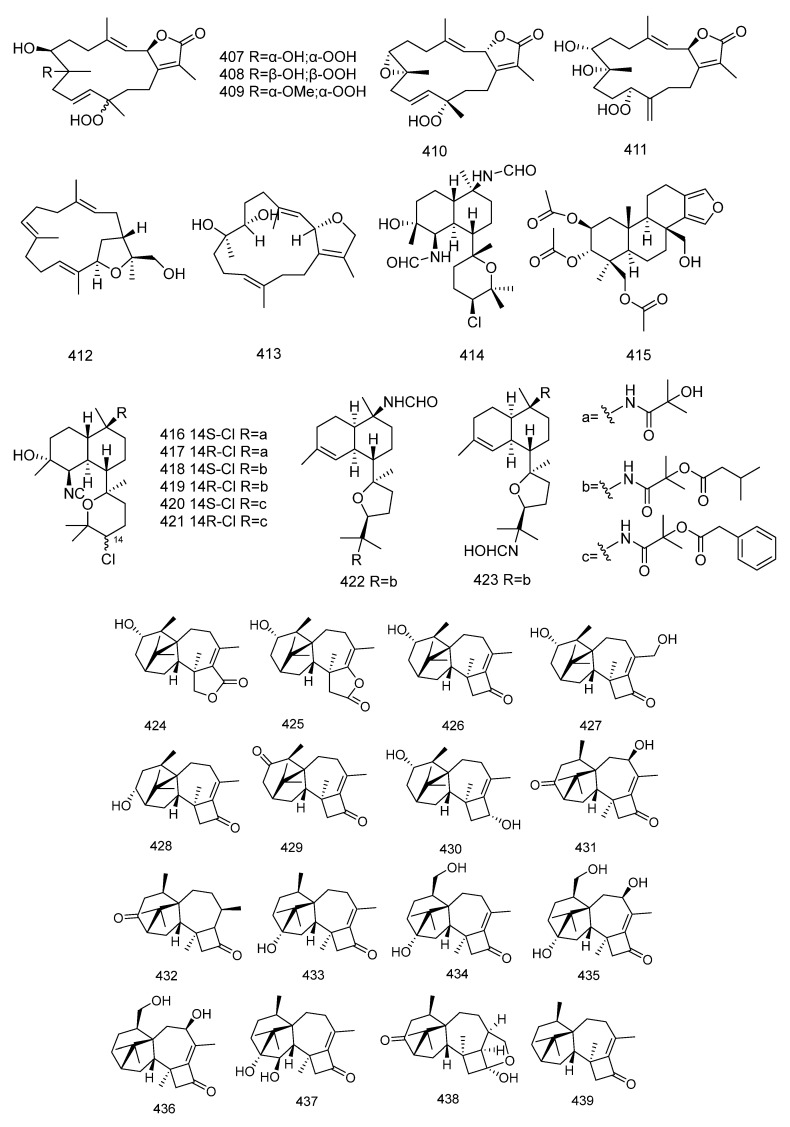

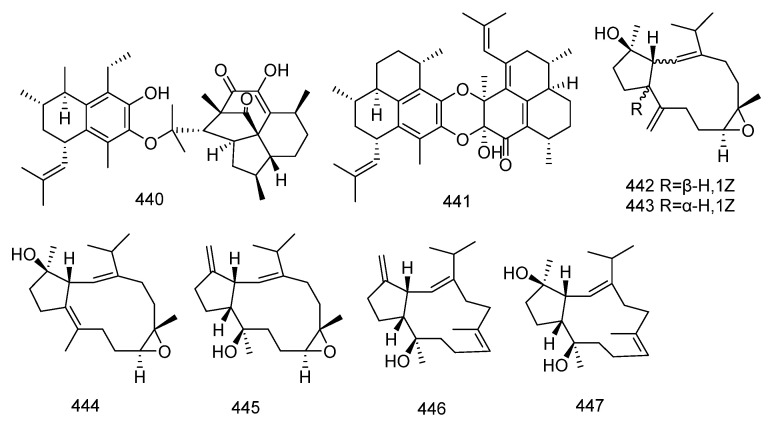
Chemical structures of sesquiterpenoids (**305**–**447**).

In 2022, Misaki Nagasaka and colleagues isolated a new furanocembranoid diterpene, 11-hydroxy-Δ^12^(^13^)-pukalide (**311**), from the Okinawan soft coral *Sinularia* sp. The compound exhibited toxicity in the brine shrimp lethality assay [79]. The chemical structures of Clerodane Diterpenes are depicted in Figure 8.

#### 2.6.7. Eunicellin-Type Diterpenes (**312**–**315**)

In 2020, three eunicellin-type diterpenes, namely briarellin T, asbestinin 27, asbestinin 28, and a known compound asbestinin 17 (**312**–**315**) were isolated from the octocoral *Briareum asbestinum*. The structures of these novel compounds were determined through extensive NMR analysis and HRMS [80]. The chemical structures of Eunicellin-type diterpenes are depicted in Figure 8.

#### 2.6.8. Lobane Diterpenoids (**316**–**333**)

Lobane-type diterpenoid compounds are not commonly found in marine soft corals. In 2020, Fei YE and their colleagues isolated three new Lobane-type diterpenoid compounds, 13-methoxyloba-8,10,15(16),17(18)-tetraene, 8,10,13(15)Z,16E-lobatetraene and 19-hydroxy- lobatetraene, along with a new natural compound, 17,18-epoxyloba-16-acetoxy- 8,10,13(15)-trien (**316**–**319**) from the soft coral *Sinularia polydactyla* collected in the South China Sea. Additionally, a known related diterpenoid compound, 18-methoxyloba-8,10,13(15),16(17)-tetraene (**320**) was also isolated. The structures of the three new compounds were determined through extensive spectroscopic analysis and literature research [81].

In 2022, Kosuke Sato and their colleagues isolated a new lobane-type diterpenoid, loba-8,10,13(15)-triene-14,17,18-triol 17-acetate (**321**), from the Okinawan soft coral *Lobophytum* sp. Additionally, they isolated five known compounds: lobatriene (**322**), (17R)-loba-8,10,13(15)-triene-17,18-diol (**323**), loba-8,10,13(15)-triene-14,17,18-triol 14,17-diacetate (**324**), loba-8,10,13(15)-triene-14,17,18-triol 14-acetate (**325**), and lobatrienetriol (**326**). The structures of these compounds were determined by extensive spectroscopic methods, including NMR and HRMS [82].

In 2023, seven new lobane-type diterpenoid compounds, lobocatalens A-G (**327**–**333**) were isolated from the soft coral *Lobophytum catalai* collected in the Xisha Islands. The structures and absolute configurations of these compounds were determined through extensive spectroscopic analysis, comparisons with literature data, QM-NMR, and TDDFT-ECD [83]. The chemical structures of lobane diterpenoids are depicted in Figure 8.

#### 2.6.9. Diterpene Derivative (**334**–**386**)

In 2019, two new sponge diterpenoid derivatives, 15α,16α-dimethoxy-15,16- dihydroepispongiol and 15α-ethoxyepispongiol-16(15H)-one (**334**–**335**) were isolated from the sponge *Spongia officinalis* collected in the South China Sea. Additionally, three known analogs (**336**–**338**) were also obtained [29]. In the same year, Xingwang Zhang and colleagues isolated three C17γ-lactonenor diterpenoids (**339**–**341**) and notable C25 manoalide-type esterterpenoids (**342**–**347**) from the sponge *Cacospongia* sp. collected in the South China Sea. Among them, compounds **341a/341b** (an inseparable enantiomer pair), **342a**, **343a**, and **344** were new compounds. The structures and absolute configurations of these new compounds were determined through HR-ESI-MS and NMR data analysis, optical rotation comparisons, and experimental and calculated ECD and Mo_2_(OAc)_4_-induced Circular Dichroism (ICD) methods. The enantiomers of compounds **339**–**343** were further separated by chiral High-Performance Liquid Chromatography (HPLC) [84].

In 2022, diterpenoid derivatives Neocucurbols A-D (**348**–**351**) were isolated from the marine-derived fungus *Neocucurbitaria unguis-hominis*. These compounds contain a complex 6/6/5/5/6 polycyclic system featuring a tetrahydrofuran bridge. Neocucurbols E−H (**352**–**355**) are diterpenoids with a 6/8/6 tricyclic system. The structures of these compounds were determined through extensive spectroscopic analysis, X-ray diffraction studies, and ECD calculations [85]. In the same year, Cili Wang and colleagues isolated five new furanobutenolide-derived C19-norcembranoid diterpenes, sinudenoids A-E (**356**–**360**) from the soft coral *Sinularia densa*. Sinudenoid A (**356**) possesses a rare 5/5/11 fused tricyclic ring system. Sinudenoids B–D (**333**–**335**) share the same 5/5/6/6 tetracyclic ring system but represent two novel skeletal types. Sinudenoid E (**360**) is the second compound with a rare 8/8 bicyclic carbon core and exhibits potent anti-inflammatory activity in a zebrafish model [86]. Sergey A. Dyshlovoy and colleagues isolated six sponge diterpenes, including a new spongionellol A and five known molecules (**361**–**366**) from the sponge *Spongionella* sp. The structures of these compounds were determined through extensive MS and NMR spectroscopic analysis, as well as comparisons with literature data [87].

In 2023, five new enolic diterpenes, including three rare nitrogen-containing derivatives dictyolactams A–B (**367**–**368**) and 9-demethoxy-9-ethoxyjoalin (**369**), a rare diterpene with a cyclobutanone structure 4-hydroxyisoacetylcoriacenone (**370**) and 19-O-acetyldictyodiol (**371**) were isolated from the brown alga *Dictyota coriacea* collected in the East China Sea. Additionally, 15 known analogs (**372**–**386**) were obtained. The structures of the new diterpenes were determined through extensive spectroscopic analysis and theoretical ECD calculations [88]. The chemical structures of diterpene derivatives are depicted in Figure 8.

#### 2.6.10. Casbane-Type Diterpenoids (**387**–**397**)

In 2021, eleven casbane-type diterpenoids, named sinucrassins A–K (**387**–**397**) were isolated from the Hainan soft coral *Sinularia crass*. The structures of these compounds were determined through extensive spectroscopic analysis and comparison with data in the literature. The absolute configurations of the compounds were established through X-ray diffraction analysis and electronic circular dichroism calculations [89]. The chemical structures of casbane-type diterpenoids are depicted in Figure 8.

#### 2.6.11. Cembranoid Diterpene (**398**–**413**)

The cembrane-type diterpenes are among the marine diterpenoid compounds isolated from marine organisms [90]. Characterized by a 14-carbon ring skeleton containing five-, six-, seven- or eight-membered lactone rings. The cembrane-type diterpenes have attracted significant research interest from developers due to their unique structural properties and biological activities.

In 2019, Chin-Soon Phan and colleagues isolated a new cembrane-type diterpenoid, sinulaflexiolide P (**398**), from the soft coral *Sinularia flexibilis* collected in Borneo. The structure of the compound was determined based on extensive NMR and HR-ESI-MS spectral data analysis [91]. In the same year, Kazuki Tani and colleagues isolated a new cembranolide diterpene, sarcophytonolide V (**399**), from the soft coral *Sarcophyton* sp. collected in Borneo. Additionally, they isolated six known compounds: isosarcophytonolide D (**400**), (4Z,8S*,9R*,12E,14E)-9-hydroxy-1-(prop-1-en-2-yl)-8,12-dimethyl-oxabicyclo[9.3.2]-hexadeca-4,12,14-trien-18-one (**401**), (7E,11E)-3,4-epoxy-7,11,15-cembratriene (**402**), (1S*,3S*,4S*,7E,11E)-3,4-epoxy-13-oxo-7,11,15-cembratriene (**403**), (−)-eunicenone (**404**), and 2-[(E,E,E)-7′,8′-epoxy-4′,8′,12′-trimethylcyclotetradeca-1′,3′,11′-trienyl]propan-2-ol (**405**). The structures of these compounds were determined by NMR and HRESIMS data [92].

In 2021, a novel cembrane diterpene (**406**) was isolated from the total methanol extract of the soft coral *Sarcophyton trocheliophorum*. The structure of the novel diterpenoid compound **406** was determined through extensive spectroscopic data analysis [93]. In the same year, Tarik A. Mohamed and colleagues isolated five highly oxidized cembrane diterpenoid compounds, named sarcoconvolutum A–E (**407**–**411**) from the Red Sea soft coral Sarcophyton convolutum. The structures of these compounds were elucidated using spectroscopic methods including Fourier Transform Infrared Spectroscopy (FTIR), 1D and 2D NMR, and HRMS [94].

In 2022, Takahiro Ishii and colleagues isolated a novel cembrane diterpenoid, odosinularol (**412**), from the Japanese soft coral *Sinularia* collected on the main island of Okinawa. The structure of the compound was determined by spectroscopic analysis, including one-dimensional and 2D NMR, HRESIMS, and IR spectroscopy [95].

In 2024, Jyh-Horng Sheu and colleagues isolated a natural dihydrofuran cembranoid compound, (7S,8R)-dihydroxydeoxyaplysinopsin (**413**), from the octocoral *Sarcophyton stellatum*. The absolute configuration of the compound was determined by single-crystal X-ray diffraction analysis, and its structure was confirmed by 2D NMR and comparison with literature data [96]. The chemical structures of cembranoid diterpene are depicted in Figure 8.

#### 2.6.12. Acetoxy Diterpene (**414**–**423**)

In 2019, one new naturally occurring nitrogen-containing kalihinane-type diterpenoid, bisformamidokalihinol A (**414**) was isolated from the nudibranchs *Phyllidiella pustulosa*, *Phyllidia coelestis*, as well as their sponge prey *Acanthella cavernosa*, collected in the South China Sea. The structure of the compound was determined through extensive spectroscopic analysis and comparison with spectral data reported in the literature [97].

In 2021, a novel acetoxy diterpenoid compound, 2β,3α,19-Triacetoxy-17- hydroxyspongia- 13(16),14-diene (**415**) was isolated from the sponge *Spongia officinalis Linnaeus*. The structure of the compound was elucidated through extensive spectroscopic analysis, quantum chemical calculations of NMR parameters, and ECD analysis. The compound exhibited cytotoxic activity against the K562 cell line with a half-maximal inhibitory concentration (IC_50_) value of 7.3 micromolar per liter [98].

In 2022, eight new α-acyloxy amide substituted kalihinane diterpenes, named kalihiacyloxyamides A–H (**416**–**423**) were isolated from the sponge *Acanthella cavernosa* collected in the South China Sea. The planar structures and absolute configurations of these compounds were determined through extensive 1D and 2D NMR, HRESIMS analysis, single-crystal X-ray diffraction, and CD spectroscopy analysis [99]. The chemical structures of acetoxy diterpene are depicted in Figure 8.

#### 2.6.13. Harziane-Type Diterpene (**424**–**439**)

In 2019, two new harziane diterpene lactones, harzianelactones A and B (**424**–**425**) were isolated from the soft coral-derived fungus *Trichoderma harzianum*. These two novel compounds possess a fused 6/5/7/5 carbon ring core and contain a lactone ring system. Additionally, five new harziane diterpenes, harzianones A-D and harziane (**426**–**430**) were also isolated. The structures of these compounds were determined through extensive NMR spectroscopic analysis, ECD calculations, Optical Rotatory (OR) measurements, and X-ray diffraction [100].

In 2021, five new harziane-type diterpene derivatives, harzianol K, harzianol L, harzianol M, harzianol N and harzianol O (**431**–**435**) were isolated from the deep-sea sediment-derived fungus *Trichoderma* sp. SCSIOW21. Additionally, two known compounds, hazianol J and harzianol A (**436**–**437**) were also obtained. The relative configurations of the compounds were determined through extensive 1D and 2D NMR spectroscopy and HR-ESI-MS. The absolute configurations of the compounds were established through ECD curve calculations and X-ray crystallographic analysis [101].

In 2023, one new diterpene, harziaketal A (**438**), along with one known diterpene, harzianone (**439**) were isolated from the culture of a marine algae-epiphytic fungus *Trichoderma* sp. The structures and relative configurations of the compounds were determined through NMR and HR-ESI-MS data. The absolute configuration of harziaketal A (**438**) was determined through X-ray diffraction. Notably, harziaketal A features a hemiketal unit within the four-membered ring of a harziane-type diterpene, which is a first-time discovery in this class of compounds [102]. The chemical structures of harziane-type diterpene are depicted in Figure 8.

#### 2.6.14. Bis-Diterpenes (Diterpene Dimers) (**440**–**441**)

In 2022, two novel asymmetric diterpenoid compounds, biselisabethoxane A and biselisabethoxane B (**440**–**441**) were isolated from the gorgonian coral *Pseudopterogorgia elisabethae*. Biselisabethoxane A is the first marine-derived C40 dimer composed of two different diterpenoid fragments, while biselisabethoxane B is a fused heterodimer coupled from two amphilectane-based fragments. The structures of these compounds were elucidated through extensive 1D and 2D NMR spectroscopic data analysis. The molecular structure of biselisabethoxane A was further confirmed by X-ray crystallography [103]. The chemical structures of bis-diterpenes are depicted in Figure 8.

#### 2.6.15. Capnosane Diterpenes (**442**–**447**)

In 2022, five new capnosane diterpenes, sarboettgerins A-E (**442**–**446**) were isolated from the soft coral *Sarcophyton boettgeri* collected in the South China Sea. Additionally, one known related compound, pavidolide D (**447**) was also obtained. The structures of these compounds were determined through extensive spectroscopic analysis, ^13^C NMR, and X-ray diffraction [104].

Furthermore, many marine diterpenoid compounds can vary among populations. For example, Ana Débora Nunes Pinheiro, Erick Alves Pereira Lopes-Filho, and their colleagues compared the diterpenoid variations among three populations of the brown alga Dictyota mertensii, further emphasizing the necessity of conducting temporal and geographical variation studies to establish the chemical classification boundaries of this species [105]. The chemical structures of capnosane diterpenes are depicted in Figure 8.

## 3. Diterpenoids Biological Activities of Marine-Derived Diterpenoids

Marine-derived diterpenoid compounds exhibit complex structures and diverse types, possessing a wide range of biological activities. They are often used in the treatment of cancer and inflammatory diseases. These compounds also demonstrate antitumor, anti-inflammatory, antibacterial, antiviral, antioxidant, and antifouling activities. This article systematically reviews the research on the activities of marine-derived diterpenoid compounds.

### 3.1. Antiviral Activity

Many infectious diseases become epidemics due to viral infections. For example, the recent novel coronavirus pneumonia is caused by the new SARS-CoV-2 virus. The spread of viruses not only has a direct impact on people’s health but may also have adverse effects on the economy, society, and political stability. Therefore, the development of antiviral drugs is a direction that researchers are striving for. One of the widely concerned antiviral compounds is diterpenes, which can be obtained from the ocean.

In 2019, Cintia Lhullier and colleagues conducted activity tests on the compounds raspailol and raspadiene from the sponge *Raspailia bouryesnaultae*, which are clerodane diterpenes. The experiment indicated that raspadiene inhibited viral HSV-1 (KOS strain) replication by 83%, and raspadiene also inhibited HSV-1 (29R strain) replication by more than 70%. Further plaque reduction assays for the compound raspadiene showed that it exhibited the best activity, with both tested strains having an SI value greater than 3.0 [78]. Oxalierpenes A and B (**73**–**74**) exhibit antiviral activity against the H1N1 virus and respiratory syncytial virus (RSV), with IC_50_ values ranging between 2.8 and 9.4 μM [41]. Compounds **75** and **76** demonstrate antiviral activity against influenza A virus (IAV) strains A/WSN/33 (H1N1) and A/PR/8/34 (H1N1), with IC_50_ values ranging from 12.6 to 46.8 μM [42]. Compounds **201**–**212** were tested for cytotoxic activity against several cancer cell lines. Among them, compound **211** showed moderate activity against the PC3 and PBL-2H3 cell lines, with half-maximal inhibitory concentration (IC_50_) values of 24.8 μM and 27.2 μM, respectively [59]. The potential anti-proliferative effects of compounds **305**–**310** on the human lung cancer cell line A549 were evaluated, and it was observed that diterpenes with a hydroxyl group at the C-6 position exhibited moderate cytotoxicity activity, with half-maximal inhibitory concentration (IC_50_) values below 25 µM. The potential antiviral activity against herpes simplex virus type 1 (HSV-1, KOS, and 29R strains) was assessed, and the novel compound **306** showed the best performance, with inhibition rates of 83% and 74% against HSV-1 (KOS and 29R strains) replication, respectively [78]. Compounds **424**–**430** exhibited potent phytotoxicity against the seedling growth of amaranth and lettuce. Harziane diterpenes, known for their remarkable bioactivities, are rarely reported, and this is the first report studying the phytotoxicity of harziane diterpenes, providing a new application for such compounds in agriculture for future research [100]. Compounds **416**–**423** were tested for cytotoxicity against various human cancer cell lines, including the leukemia K562, normal liver L-02, pancreatic cancer ASPC-1, lung cancer H69AR, and breast cancer MDA-MB-231 cell lines [106,107]. The bioactivity results showed that compounds **418** and **421** exhibited moderate cytotoxicity against the K562 cell line, with IC_50_ values of 6.4 and 6.3 μM, respectively. Compounds **421** and **423** displayed IC_50_ values of 7.3 and 7.9 μM against the MDA-MB-231 cell line, respectively. Compounds **407**–**411** were tested for cytotoxicity against lung adenocarcinoma, cervical cancer, and oral cancer (A549, HeLa, and HSC-2 cell lines, respectively), and the most cytotoxic compound **410** showed activity against the A549 and HSC-2 cell lines, with IC_50_ values of 49.70 and 53.17 μM, respectively [94].

Many diterpenes derived from marine natural products have shown anti-HIV properties. In 2019, Karlo Wittine, Lara Saftíc, Željka Peršuríc, and Sandra Kraljevíc Pavelíc researched the antiviral activity of marine structures, including marine diterpenes. The mechanisms of action include blocking different stages of the HIV-1 replication cycle, such as reverse transcriptase inhibitors, protease inhibitors, or entry inhibitors. Among them, diterpenes from marine algae have become a hot topic of research due to their promising anti-HIV activity [108]. Pardo-Vargas et al. characterized three new diterpenes, dolabelladienols A-C, isolated from the Brazilian northeast marine brown algae *Dictyota pfaffii*. The new compounds dolabelladienols A and B showed potent anti-HIV-1 activity, with IC_50_ values of 2.9 and 4.1 μM, and low cytotoxic activity against MT-2 lymphocyte tumor cells. These anti-HIV-1 drugs are even more active than the previously known 2,6-dolabelladienes series [109]. Caroline de Souza Barros et al. tested the anti-HIV-1 activity of marine dolastanes and secodolastane diterpenes isolated from the brown algae *Canistrocarpus cervicornis* [110]. They found that marine diterpenes inhibited HIV-1 replication in a dose-dependent manner without exhibiting cytotoxicity. Stephens and partners. examined 8,10,18-trihydroxy-2,6-dienediene pretreated in peripheral blood mononuclear cells (PBMCs) and macrophages, as well as its protective effects in a cervical explant model in vitro [111]. Pretreatment of PBMCs and macrophages with dolabelladienetriol demonstrated inhibition of HIV-1 replication. Furthermore, in the explant model, dolabelladienetriol inhibited viral replication in a dose-dependent manner, without compromising tissue viability [112]. Although the dolabellane diterpenes from the brown alga *Dictyota* sp. exhibited strong anti-HIV-1 activity, this was not confirmed for dolabellane diterpenes isolated from octocorals. Therefore, several chemical transformations have been conducted [113]. For example, Oxygenated dolabellanes derivatives obtained through the epoxidation of keto-trienes, ring opening of epoxides, and allylic oxidation exhibited significantly improved antiviral activity and low cytotoxicity against MT-2 cells [108].

In 2023, Ana Débora Nunes Pinheiro Georgii and Valéria Laneuville Teixeira conducted research on diterpenoids derived from brown algae of the genera *Dictyota* and *Canistrocarpus*, screening for active diterpenoid compounds among 26 different diterpenoids, with the most prominent antiviral activity observed [114]. Their investigation revealed the structural diversity of diterpenoids produced by algae in the *Dictyoteae* family. These metabolites have demonstrated significant in vivo activity and are involved in various critical biological processes, playing distinct and important roles in the marine environment [115]. Furthermore, studies have shown that these compounds play roles in chemical defense systems against herbivores, resistance to biofouling, and allelopathy [116,117]. The bioactivities of diterpenoids produced by Dictyoteae algae also include substances with antitumor, antibacterial, and antifungal effects [118]. Various diterpenoid compounds have been isolated from *Dictyota menstrualis*, which contains secondary metabolites with anti-HIV (Human Immunodeficiency Virus) and anti-HSV (Herpes Simplex Virus) properties. Research on these metabolites aids in studying their mechanisms of action and potential applications in the development of antiviral therapies [119,120]. D. menstrualis is capable of producing pachydictyol A and isopachydictyol A, both of which exhibit antiplatelet and anticoagulant activities, as well as having antileishmanial effects [121,122]. Two dolastane-type diterpenoids, 4-hydroxy-9,14-dihydroxydolasta-1(15),7-diene and 4,7,14-trihydroxydolasta-1(15), 8-diene isolated from Canistrocarpus cervicornis, exhibited antiviral activity against HSV-1 [4]. They inhibited HSV-1 infection in Vero cells, suggesting their potential as antiviral drugs.

The marine-derived sesquiterpenoids from the soft coral Lobophytum species have demonstrated a variety of biological activities, including antiviral [123], immunostimulatory [124], anti-inflammatory [125], anticancer [126], and antibacterial activities [127]. In 2020, Yichang Liu et al. [128] found studies reporting that sarcophytolides, including 13-acetoxysarcophytolide, sarcophytolide M, and 14-deoxyiso-sarcophytolide, exhibited potent cytotoxic effects on leukemia and lymphoma cells, with half-maximal inhibitory concentration (IC_50_) values ranging from 1.2 to 7.1 μM [129]. Research has found that 13-AC, derived from the soft coral Sarcophyton crassocaule, has broad cytotoxic effects on leukemia P388 cells [130], female bladder transitional cell carcinoma (BFTC) cells [131], and human gastric adenocarcinoma (AGS) gastric cancer cells [132]. In addition, Sinulariaone A (**252**) showed cytotoxicity towards human promyelocytic leukemia HL-60 cells, with an IC50 value of 38.01 μM [69].

### 3.2. Anti-Inflammatory Activity

The pathogenesis of many diseases is closely related to inflammation, and marine diterpenoid compounds have demonstrated significant anti-inflammatory properties, suggesting their potential therapeutic efficacy in a range of diseases including neuritis, colitis, and atherosclerosis. The kahukuene B (**22**) exhibited significant anti-inflammatory activity, with an IC_50_ value of 6.66 μM [26]. The compound 5,5,6,6,5-pentacyclic diterpene, sponalactone (**52**) was tested for its in vitro anti-inflammatory activity. In preliminary assays, the compound exhibited inhibitory effects on lipopolysaccharide-induced nitric oxide production in RAW264.7 macrophages, with IC_50_ values ranging from 12 to 32 μM/L [33]. To discover bioactive lead compounds, Chi-Jen Tai and colleagues conducted activity tests on 17-dehydroxysponalactone (**53**), including assays to determine the compound’s inhibitory activity against superoxide anion generation and elastase release in human neutrophils induced by formyl-methionyl-leucyl-phenylalanine/cytochalasin B (fMLF/CB), as well as its cytotoxicity against three tumor cell lines (mouse leukemia P388, human cholangiocarcinoma HuCCT, and human colorectal adenocarcinoma DLD-1) and one human skin fibroblast cell line (CCD-966SK). The results indicated that 17-dehydroxysponalactone (**53**) exhibited good anti-inflammatory activity [36]. Cytotoxicity, antibacterial, and anti-inflammatory activity assays were conducted on compounds **54**–**56**. The results revealed that all three compounds were able to inhibit the production of superoxide anions in human neutrophils stimulated with fMLF/CB. Additionally, *Staphylococcus aureus* growth inhibition experiments showed that compound **54** had inhibition rates of 46%, 47%, and 93% at concentrations of 50, 100, and 200 µM, respectively, while compound **55** had inhibition rates of 24%, 42% and 40% at the corresponding concentrations. Therefore, compared to compound **55**, compound **54** exhibited stronger inhibitory effects on the growth of *Staphylococcus aureus* [37]. Compound **77** as a non-competitive inhibitor of protein tyrosine phosphatase 1B (PTP1B), had a half maximal inhibitory concentration (IC_50_) value of 8.60 ± 0.53 micromolar (µM). Compound **93** demonstrated significant α-glucosidase inhibitory activity with an IC_50_ value of 19.96 ± 0.32 µM. Furthermore, compounds **80**, **84**, and **98** strongly inhibited the production of nitric oxide in lipopolysaccharide-stimulated RAW264.7 macrophages [43]. In activity assays, sarcomililatols D (**121**) exhibited potent tumor necrosis factor TNF-α inhibitory activity (IC_50_ = 6.1 μmol/L), superior to the positive control drug dexamethasone (IC_50_ = 8.7 μmol/L), and showed no significant cytotoxicity towards RAW264.7 cells, with a CC_50_ value exceeding 50 μmol/L. This suggests that sarcomililatols D (**121**) can serve as a model compound for the development of novel and promising anti-inflammatory lead compounds or drug candidates [48]. The anti-inflammatory activity of compounds trichodermanins F-H (**195**–**197**) was evaluated, and in terms of anti-inflammatory activity against LPS-induced NO production, compound **195** demonstrated significant inhibitory efficacy with an IC_50_ value of 2.19 ± 0.25 μmol/L, which was three times lower than the positive control indomethacin (IC_50_ = 8.76 ± 0.92 μmol/L). Further Western blot and immunofluorescence experiments confirmed that the mechanism of action was the inhibition of the NF-κB activation pathway by compound **195**, highlighting it as a promising starting point for the development of novel anti-inflammatory drugs [57]. Anti-inflammatory activity tests were conducted on compounds **312**–**315**, and researchers used the sulforhodamine B (SRB) assay to detect the survival rate of THP-1 cells and tested concentrations up to 50 µM. Compared to unstimulated control cells, LPS led to a significant increase in the production of TNF-α, interleukin IL-6, and IL-1β in THP-1 cells. These molecules are potent pro-inflammatory cytokines produced by immune cells at the site of inflammation and play crucial roles in local and systemic inflammatory responses [133]. Cytokine IL-8 is a potent neutrophil chemoattractant that also regulates angiogenesis and metastasis [134]. The pretreatment of cells with compounds **312**–**315** also significantly reduced cytokine levels; these anti-inflammatory effects were more pronounced at a concentration of 50 µM. Additionally, it has been reported that inducible enzyme cyclooxygenase-2 (COX-2) plays a key role in inflammatory responses by overproducing pro-inflammatory prostaglandins such as prostaglandin E2 (PGE2) [135]. Experiments showed that compounds **312**–**315** significantly reduced the production of pro-inflammatory cytokines TNF-α, IL-6, IL-1β, and IL-8 in LPS-stimulated THP-1 macrophages and downregulated the expression of COX-2. These findings support the potential use of these marine compounds as drugs for the treatment of inflammatory diseases [135]. Hazianol J (**436**) exhibited weak anti-inflammatory activity at a concentration of 100 µM, with a NO inhibition rate of 81.8% [101]. In vitro bioassays showed that compound **447** displayed potent anti-neuroinflammatory activity against LPS-induced NO release in BV-2 microglia cells, suggesting its potential future development as a novel neuroprotective agent [104].

In addition, in 2019 Maria G. Daskalaki and her partners studied the anti-inflammatory activity of neorogioltriol, neorogioldiol, and *O*^11^,15-cyclo-14-bromo-14,15-dihydrorogiol-3,11-diol. The above compound can suppress macrophage activation, promote an M2-like anti-inflammatory phenotype, and suppress iNOS induction and nitric oxide production [136]. In 2024, Nguyen Viet Phong and colleagues evaluated the inhibitory activity of ten cembranoid diterpenes, sinumaximols A-H, sethukarailin, and 5-epinorcembrene, isolated from the soft coral Sinularia maxima, against soluble epoxide hydrolase (sEH). Compounds sinumaximols C and sethukarailin exhibited significant sEH inhibition with IC_50_ of 70.68 µM and 78.83 µM, respectively. Enzyme kinetic analysis revealed that these two active compounds inhibited sEH through a non-competitive mode. Furthermore, computer simulation methods, including molecular docking and molecular dynamics simulations, confirmed their stability and interaction with sEH, highlighting their potential as natural therapeutic agents for managing cardiovascular and inflammatory diseases [137].

### 3.3. Antibacterial Activity

Some marine diterpenoid compounds exhibit a broad antibacterial spectrum, low toxicity, and high antibacterial activity. Compounds **108**–**113** exhibit antibacterial activity against Gram-positive pathogens such as Staphylococcus aureus, Streptococcus pneumoniae, and *Enterococcus faecalis*, with (+)-10-epiagelasine B (**109**) demonstrating the strongest activity, with a minimum inhibitory concentration (MIC) ranging from 1 to 8 µg/mL [46]. *Nephthea* sp. possesses the biosynthetic capability to produce compounds containing α-methylene-γ-lactone groups and cembranes with isopropyl functional groups. Although compounds **114**–**119** all exhibit antifungal activity, compounds **114** and **119** show relatively better inhibitory activity against the mycelial growth of L. thermophilum, with MIC values of 12.5 µg/mL [47]. Situmulins B (**148**) not only exhibits strong antibacterial activity against fish pathogenic bacteria *Streptococcus parauberis* FP KSP28 and Photobacterium damselae FP2244, with both having a 90% minimum inhibitory concentration (MIC_90_) of 25 micromolar but also demonstrates significant inhibitory effects on vancomycin-resistant Enterococcus faecium strains G1, G4, G7, G8, and G13 from multiple individuals, with MIC_90_ values of 25, 50, 100, 50 and 25 micromolar, respectively [51]. Antibacterial experimental results indicate that talascortene I (**254**) exhibits significant activity against the pathogenic fungus Curvularia spicifera, with an MIC value of 1 µg/mL, providing experimental evidence for the development of talascortene I (**254**) as a potential lead compound for biopesticides [70]. Compound **269** exhibits strong antifungal activity against *Candida albicans* ATCC10231, with an MIC value of 2.9 µM. The differences in antifungal activity among compounds **265**–**271** suggest that the glycosyl moiety, the length of the fatty acid side chain, and the C-2 carboxylic acid may influence antifungal activity [73]. Compounds **282**–**290** were evaluated for antibacterial activity, cell growth inhibition of A549, Hela, HepG2, and CCRF-CEM cancer cell lines, and inhibition of lipopolysaccharide (LPS)-induced nitric oxide (NO) production in RAW264.7 macrophages. The antibacterial activities were evaluated with the broth dilution assay [138]. The results showed that compounds **282**, **283,** and **285**–**287** exhibited antibacterial activity against *Staphylococcus aureus* and *Bacillus subtilis* (with MIC ranging from 4 to 16 µg/mL) [75]. Sarcophytonolide V (**399**) exhibited inhibition against the hyphal growth of *Ochroconis humicola* and *Haliphthoros milfordensis* at MIC 6.25 µg/mL [92].

### 3.4. Antineoplastic Activity

Ceylonamide G (**104**) inhibits the growth of human prostate cancer cells DU145 in two-dimensional monolayer cultures with a half-maximal inhibitory concentration (IC_50_) of 6.9 µM. Based on changes in spheroid morphology, the minimum effective concentration of Ceylonamide G (**104**) is 10 µM. Ceylonamide G (**104**) is expected to be effective in tumor xenograft mouse models. Therefore, they hold promise as new drug candidates for cancer chemotherapy [44]. (−)-agelamide D (**107**) enhances the radiosensitivity of Hep3B cells, manifesting as decreased clonogenic ability and increased apoptotic cell death. Additionally, (−)-agelamide D elevates the expression of protein kinase RNA-like endoplasmic reticulum kinase/eukaryotic translation initiation factor 2α/activating transcription factor 4 (PERK/eIF2α/ATF4) in multiple HCC cell lines, which is a key pathway of the unfolded protein response (UPR), and augments radiation-induced UPR signaling. In vivo xenograft experiments confirm that (−)-agelamide D potentiates the inhibitory effect of radiotherapy on tumor growth without causing systemic toxicity. Immunohistochemical results show that (−)-agelamide D further increases radiation-induced ATF4 expression and apoptotic cell death, which is consistent with our in vitro findings [45]. Bioactivity assessments reveal that sarcoxacyclols A (**127**) exhibit significant tumor necrosis factor (TNF)-α inhibitory activity (IC_50_ = 9.5 µmol/L). Preliminary molecular docking studies suggest that hydroxyl and acetoxy groups play crucial roles in the computationally predicted binding modes of the diterpenes with the protein [49]. Bioactivity assays show that compounds **171**–**185** and **191** inhibit the proliferation of human non-small cell lung cancer cell lines H1975, H1299, and A549, as well as the human breast cancer cell line MDA-MB-231, with half maximal inhibitory concentration (IC_50_) values ranging from 1.92 to 35.63 µM. Among them, compounds **173**, **180**, and **183** demonstrate significant antiproliferative activity with IC_50_ values of 1.92–8.82 µM. Molecular mechanism studies indicate that the antitumor activity of compound **173** is closely related to the modulation of ROR1 and ErbB3 signaling pathways [55].

In 2021, Abel M. Forero and colleagues screened three marine diterpenoid compounds that interact with tubulin and induce cell death through apoptosis. This study characterized the interaction between these compounds and tubulin using nuclear magnetic resonance (NMR) spectroscopy and proposed their binding modes through docking studies and molecular dynamics simulations [139]. Firstly, a library of 32 natural and semi-synthetic marine diterpenoid compounds isolated from four octocoral species: Eunicea, Plexaura, Pseudoplexaura, and Erythropodium, was constructed [140,141,142,143,144,145]. Among them, 12 compounds possess the briarane nucleus, 17 have the kaurane nucleus, and 3 bear the polyacetylene nucleus. The MTT assay was employed to determine the cell viability of these compounds against three human cancer cell lines (A549 human lung cancer, MCF7 human breast adenocarcinoma, and PC3 human prostate cancer) [146]. Notably, three compounds exhibited significant cytotoxic activity against these three human cancer cell lines. Flow cytometry analysis was conducted on the three most potent compounds to investigate their apoptosis-inducing effects on the A549 cell line. NMR spectroscopy was utilized to characterize the interaction between these compounds and tubulin [147], assessing the interactions between the active diterpenoid compounds and tubulin. These interactions were characterized through computational studies of molecular docking and molecular dynamics (MD) simulations. Consequently, the three diterpenoid compounds demonstrated activity against the three human cancer cell lines, induced apoptosis in the A549 cell line, and showed interactions with tubulin, preferentially targeting the taxane-binding site.

### 3.5. Antifouling Activity

Marine fouling refers to the adherence of marine organisms, including animals, microorganisms, and plants, which tend to grow in both normal and abnormally high saline water, onto offshore equipment, leading to rapid degradation. Consequently, the research has become a significant area of study into marine natural compounds with antifouling properties [148]. The compounds **3** and **4** showed strong antifouling activity against *Mytilus galloprovincialis*, and showed 75% and 70% inhibition, respectively [18]. The marine antifouling activity tests conducted on compounds **17** and **18** revealed that both compounds exhibited potent antifouling activity against various sessile organisms [23].

Kyriakos C. Prousis and colleagues tested the antifouling activity of bromosphaerol, a diterpenoid compound isolated from the red alga Sphaerococcus coronopifolius. The results indicated that bromosphaerol is a highly promising antifouling agent, demonstrating strong anti-settlement activity against larvae of the barnacle Balanus amphitrite (also known as Amphibalanus amphitrite) with extremely low toxicity. Additionally, 15 structural analogs of bromosphaerol were successfully synthesized using different synthetic routes. The anti-settlement activity (EC_50_) and toxicity levels (LC_50_) of the bromosphaerol derivatives were evaluated using cyprid larvae and nauplii of the barnacle arthropod A. amphitrite as model organisms. Some of the derivatives exhibited varying degrees of antifouling activity, acting through non-toxic mechanisms [149]. Maria Protopapa and her partners studied the antifouling potential of 25 secondary metabolites derived from species of the genus *Laurencia* and conducted a comprehensive assessment of ecotoxicity in non-target Marine arthropod and vertebrate cell lines. The results show that only the compound perforenol has strong antifouling activity [150].

### 3.6. Others Activity

Compounds **367**–**386** all demonstrated cytoprotective effects against oxidative stress in neuronal-like PC12 cells. Among them, 18-acetoxy-6,7-epoxy-4-hydroxydictyo-19-al (**376**) exerts its antioxidant mechanism through the activation of the Nrf2/ARE signaling pathway and also shows significant neuroprotective effects against cerebral ischemia–reperfusion injury (CIRI) in vivo [88]. The information related to compound activity is shown in Table 1.

## 4. Synthesis of Marine-Derived Diterpenoids

Marine diterpenes are synthesized from four isoprene units and have a basic structure starting with the molecular formula C_20_H_32_. Many marine diterpenes undergo various modifications, such as methylation, acetylation, hydroxylation, epoxidation, and prenylation, thereby incorporating additional carbon and oxygen atoms into their molecular formulas.

### 4.1. Synthesis of Analogs of Bromosphaerol

**Figure 9:**
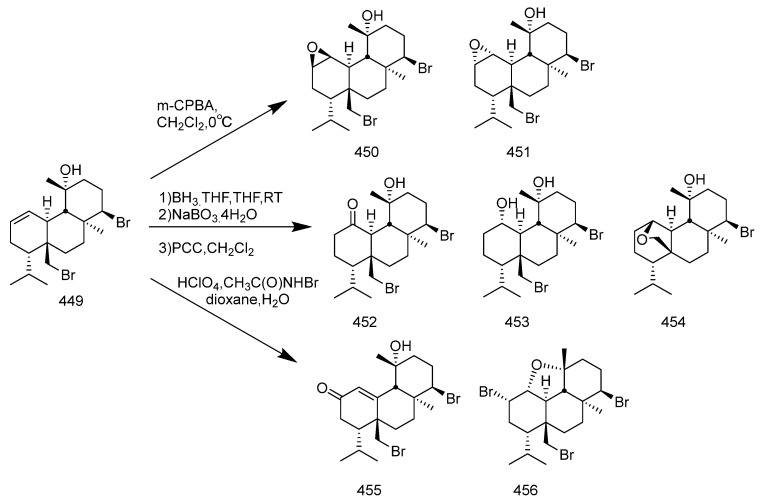
Synthesis of bromosphaerol derivatives **450**–**456**.

**Figure 10:**
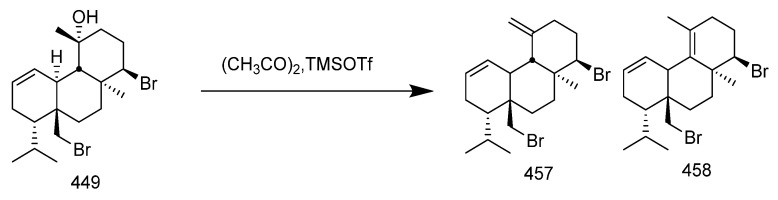
Synthesis of bromosphaerol derivatives **457** and **458**.

**Figure 11:**
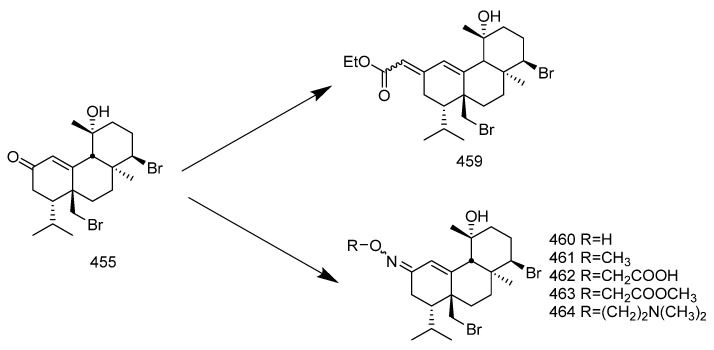
Synthesis of bromosphaerol derivatives **459** and **462**.

In 2021, Prousis and Stefanos Kikionis et al. employed various synthetic routes to successfully synthesize 15 structural analogs (**449**–**463**) of bromosphaerol (**448**) isolated from Sphaerococcus coronopifolius. These analogs featured different functional groups. Bromosphaerol (**448**) underwent a hydroboration/oxidation reaction using a tetrahydrofuran borane complex and sodium perborate, followed by pyridinium chlorochromate (PCC) oxidation of the resulting alcohols. This synthesis yielded a mixture of ketone **450** and the precursor alcohol **452**. The side product of the reaction was compound **453**, which featured an oxygen bridge between C-1 and C-17, formed through nucleophilic SN2 attack of the C-1 epimer of alcohol **453** on the brominated carbon C-17 during the hydroboration step. Treatment of bromosphaerol (**448**) with N-bromosuccinimide in a water/DMSO mixture in the presence of perchloric acid yielded α,β-unsaturated ketone **454** and C-2 brominated compound **455** (Figure 9) [149]. Furthermore, treatment of bromosphaerol (**448**) with trimethylsilyl trifluoromethanesulfonate in the presence of acetic anhydride resulted in the elimination reaction at C-11, forming isomeric alkenes bromosphaerenes B (**456**) and bromosphaerenes A (**457**) (Figure 10). Compounds bromosphaerenes B and bromosphaerenes A had been previously isolated by Fattorusso et al. [151]. Compound α,β-unsaturated ketone **454** was reacted with triethyl phosphonoacetate through the Horner-Wadsworth-Emmons reaction to produce α,β-unsaturated ester **458** with a yield of 90%. Additionally, compound **454** was treated with various substituted alkoxyamines in pyridine to yield a series of oxime derivatives (**459**–**463**) (Figure 11) [149].

### 4.2. Synthesis of Diterpene-Type Aminotriol Derivatives

In 2023, Dorottya Bai, Zsuzsanna Schelz, and their colleagues synthesized a new family of diterpenoid aminotriols derived from stevioside through a stereoselective synthetic approach [152]. The key intermediate spiro epoxide was prepared from the ester of an allyl glycol derived from steviol. The oxirane ring was opened by primary and secondary amines, resulting in a versatile library of aminotriols. The corresponding primary aminotriols were formed through palladium-catalyzed hydrogenation, and a N,O-heterocyclic compound was synthesized in a regioselective reaction. All new compounds were characterized using one-dimensional and two-dimensional NMR techniques as well as HRMS [152]. In vitro studies revealed that the aromatic N-substituted derivatives exhibited significant inhibition of cell growth in human cancer cell lines (HeLa, SiHa, A2780, MCF-7 and MDA-MB-231). The anti-proliferative activity of these compounds was assessed using the MTT method. Additionally, the introduction of an extra hydroxyl group slightly enhanced the biological activity. The drug-likeness of these compounds was evaluated through computational simulation and experimental physicochemical characterization, complemented by measurements of kinetic aqueous solubility and in vitro intestinal-specific parallel artificial membrane permeability assay (PAMPA-GI) [152].

### 4.3. Synthetic of the Diterpenes (+)-Randainin D and (+)-Barekoxide

Randainin D is a structurally unique diterpenoid due to the simultaneous presence of the trans-hydroazulenone core and the C9-butenolide moiety [153]. Bioassays showed that randainin D inhibits elastase release and superoxide-anion generation. Considering the therapeutic potential of randainin D, together with its original structure, Oleksandr Vyhivskyi and Olivier Baudoin achieve its first total synthesis and establish a general strategy toward the formation of transcycloheptane-containing polycyclic terpenes. Moreover, the developed allylation was successfully utilized in the 7-step total synthesis of (+)-barekoxide [154].

**Figure 12:**
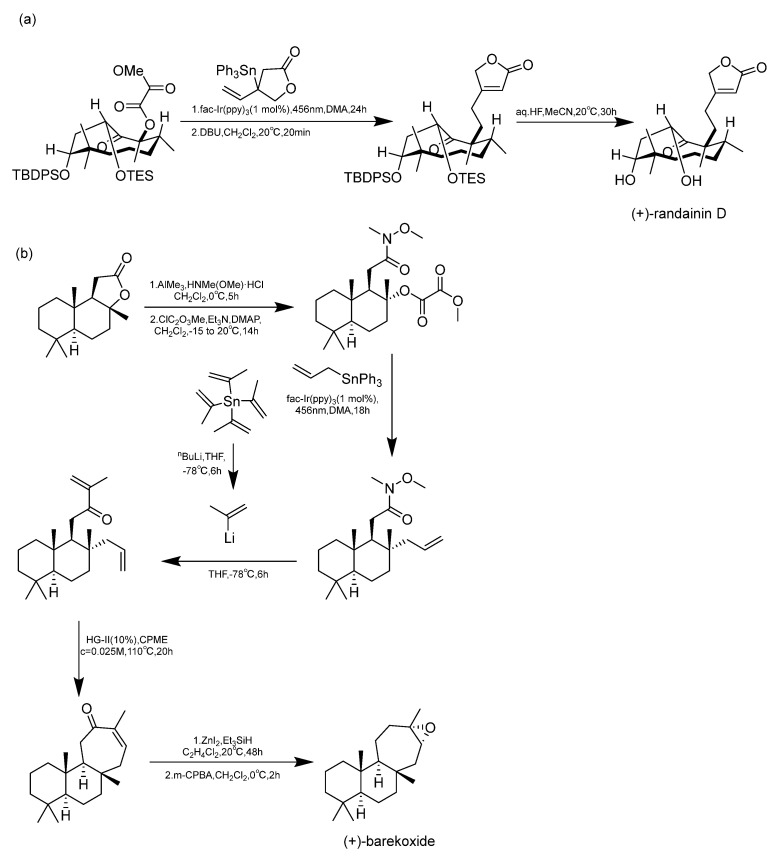
(**a**) The Total Synthetic of the Diterpenes (+)-Randainin D; (**b**) The Synthesis of (+)-Barekoxide.

Photoredox catalysis refers to the utilization of light energy to facilitate oxidation and reduction processes. Oleksandr Vyhivskyi and Olivier Baudoin achieved the enantioselective synthesis of (+)-lanostanol D and (+)-barrigtone oxide, by forming the key Cq-C(sp^3^) bonds, through Ir(III) photoredox catalysis [154]. Oleksandr Vyhivskyi and Olivier Baudoin designed the shortest synthetic route to (+)-barekoxide, starting from (+)-sclareolide [154]. Sarpong and Davies, for the first time, synthesized (+)-barekoxide through 10 steps of enantioselective synthesis starting from (+)-sclareolide [155]. These studies have enriched the synthetic strategies for constructing multicyclic systems with trans-cycloheptane frameworks, including complex terpenoids (Figure 12).

## 5. Conclusions

Natural products, with their structural diversity and extensive biological activities, are vital sources for the development of pharmaceutical intermediates and drugs. Diterpenoid compounds from marine sources, as one of the main types of marine secondary metabolites, exist in various marine organisms. Marine diterpenoids are characterized by a high degree of structural diversity, mainly due to their cyclization patterns, types and positions of functional groups, and stereochemical complexity. These structural features determine their biological activities and application potential. The synthesis of marine diterpenoids typically involves reactions such as polymerization and cyclization of isoprene units, as well as functionalization. They have a wide range of applications in the food processing, cosmetics, and pharmaceutical industries to improve human health. This review summarizes 447 marine-derived diterpenoid compounds reported in research papers published from 2019 to 2024. These marine-derived species, including corals, sponges, algae, and marine fungi, were mainly collected from mangrove forests in the South China Sea. Furthermore, this review explores the biological activities of marine diterpenoids, including anti-crototoxicity, anticancer, antibacterial, and anti-inflammatory effects. Among the 447 diterpenoids investigated, those with anti-inflammatory and cytotoxic activities are the most abundant. In terms of antibacterial activity, they exhibit antimicrobial effects against *Staphylococcus aureus*, *Bacillus subtilis*, *Streptococcus pneumoniae*, *Enterococcus faecalis*, and vancomycin-resistant Enterococcus strains. However, the complex and variable structures of marine diterpenoids pose challenges in separation and purification. With the increasing demand for bioactive diterpenoids, the extraction of diterpenoids from the ocean is far from meeting human needs. By analyzing the synthetic pathways of marine diterpenoids, they can be synthesized through these pathways. This field has tremendous potential and is crucial for resource development, medicine, and the chemical industry.

In the future, more in-depth research on marine-derived diterpenoids should be conducted. For example, further exploration of their unique chemical transformations is needed to develop more efficient synthesis methods. Additionally, efforts should be made to overcome the challenges in separation and purification to increase the yield and purity of marine diterpenoids. Moreover, the potential applications of marine diterpenoids in new fields, such as nanomedicine or environmental protection, could be investigated to fully realize their value and meet the growing demands in various industries. The development of targeted drug delivery systems using marine diterpenoids in nanomedicine could enhance their therapeutic effects and reduce side effects. In environmental protection, marine diterpenoids could be used as biodegradable surfactants or antimicrobial agents to reduce environmental pollution.

## Figures and Tables

**Table 1:** Marine-derived diterpenes with various bioactivities.

No.	Compound Name	Marine Sources	Activity	Reference
1	raspailol and raspadiene	*Raspailia bouryesnaultae*	Anti-cytotoxic activity(HSV-1, KOS and 29R)	[78]
2	oxalierpenes A and B	*Marine-fungi*	Inhibited H1N1and RSV	[41]
3	Penijanthine E and analogue	*Penicillium citrinum*	Inhibited A/WSN/33(H1N1) and A/PR/8/34(H1N1) and (IAV)	[42]
4	epoxynorspongians E	*Penicillium* sp.	Anti-cytotoxic activity(PC3 and PBL-2H3)	[59]
5	sarcoconvolutum E	*Sarcophyton convolutum*	Anti-cytotoxic activity(A549 and HSC-2)	[94]
6	dolabelladienols A and B	*Dictyota pfaffii*	Anti-cytotoxic activity(HIV-1)	[109]
7	dolastanes and secodolastane	*Canistrocarpus cervicornis*	Anti-cytotoxic activity(HIV-1)	[111]
8	pachydictyol A and isopachydictyol A	*Dictyota menstrualis*	Antiplatelet and anticoagulant activity	[121,122]
9	4-hydroxy-9,14-dihydroxydolasta-1(15),7-diene and 4,7,14-trihydroxydolasta-1(15),8-diene	*Canistrocarpus cervicornis*	Anti-cytotoxic activity(HIV-1)	[4]
10	sinulariaone A	*Sinularia*	Anti-cytotoxic activity(HL-60)	[69]
11	kahukuene B	*Laurencia majuscula*	Anti-Inflammatory Activity	[26]
12	sponalactone	*Spongia officinalis*	Anti-Inflammatory Activity	[33]
13	17-dehydroxysponalactone	*Spongia* sp.	Anti-Inflammatory Activity	[36]
14	spongenolactones A–C	*Spongia* sp.	Inhibited fMLF/CB	[37]
15	spongenolactones A	*Spongia* sp.	Inhibited *Staphylococcus aureus* activity	[34]
16	compound **93**	*Penicillium* sp.	Inhibited α-glucosidase activity	[42]
17	penpaxilloids D	*Penicillium* sp.	Anti-Inflammatory Activity	[42]
18	sarcomililatols D	*Sarcophyton mililatensis*	Anti-Inflammatory Activity	[48]
19	trichodermanins F	*Halichondria okadai*	Anti-Inflammatory Activity	[57]
20	neorogioltriol	*Laurencia*	Anti-Inflammatory Activity	[136]
21	neorogioldiol	*Laurencia*	Anti-Inflammatory Activity	[136]
22	*O*^11^,15-cyclo-14-bromo-14,15-dihydrorogiol-3,11-diol	*Laurencia*	Anti-Inflammatory Activity	[136]
23	lemnabourside E	*Lemnalia bournei*	antibacterial activity (*Staphylococcus aureus* and *Bacillus subtilis*)	[80]
24	lemnabourside F	*Lemnalia bournei*	antibacterial activity (*Staphylococcus aureus* and *Bacillus subtilis*)	[80]
25	lemnadiolboursides A-C	*Lemnalia bournei*	antibacterial activity (*Staphylococcus aureus* and *Bacillus subtilis*)	[80]
26	briarellin T, asbestinin 36 and asbestinin 37	*Briareum asbestinum*	Anti-Inflammatory Activity(inhibited TNF-α, IL-6, IL-1β and IL-8)	[80]
27	hazianol J	*Trichoderma* sp.	Anti-Inflammatory	[101]
28	pavidolide D	*Sarcophyton boettgeri*	Anti-Inflammatory	[104]
29	sinumaximols C and sethukarailin	*Sinularia maxima*	Inhibited sEH	[137]
30	(+)-10-epiagelasine B	*Agelas citrina*	Antibacterial activity(*Staphylococcus flavus*, *Streptococcus pneumoniae* and *Enterococcus faecalis*)	[46]
31	nephthecrassocolides A and nephthenol	*Nephthea* sp.	Antibacterial activity	[47]
32	situmulins B	*Sinularia tumulosa*	Antibacterial activity(*Streptococcus parauberis* FP KSP28 and Photobacterium damselae FP2244 and Enterococcus faecium G1, G4, G7, G8, G13)	[51]
33	moriniafungins E	*Curvularia hawaiiensis*	Antibacterial activity (*Candida albicans*)	[73]
34	sarcophytonolide V	*Sarcophyton* sp.	Antibacterial activity(*Ochroconis humicola* and *Haliphthoros milfordensis*)	
35	ceylonamide G	*Agelas* sp.	Inhibited Tumor Cell Growth	[45]
36	compound **171**, **180**, and **183**	*Lobophytum crassum*	Antiproliferative activity	[55]
37	9,11-dihydrogracilin A and 9,11-dihydrogracillinone A	*Dendrilla antarctica*	Antifouling Activity	[23]
38	bromosphaerol	*Sphaerococcus coronopifolius*	Antifouling Activity	[149]
39	compounds **3** and **4**	*Laurencia venusta Yamada*	Antifouling Activity (*Mytilus galloprovincialis)*	[18]
40	perforenol	*Laurencia*	Antifouling Activity	[150]

## Data Availability

Not applicable.

## Abbreviations

The following abbreviations are used in this manuscript:

MIC	Minimum inhibitory concentration
MIC_90_	90% minimum inhibitory concentration
MRSA	Methicillin-resistant Staphylococcus aureus
E. coli	Escherichia coli
XDR	Extensively drug-resistant
NMR	Nuclear magnetic resonance
MS	Mass spectroscopic
QM	Quantum mechanics
QM-NMR	Quantum mechanics-nuclear magnetic resonance
TDDFT	Time-dependent density functional theory
ECD	Electronic circular dichroism
HRESIMS	High-resolution electrospray ionization mass spectrometry
CD	Circular dichroism
NOESY	Nuclear Overhauser effect spectroscopy
HSQC	Heteronuclear single-quantum coherence
HMBC	Heteronuclear multiple bond correlation
NOE	Nuclear overhauser effect
IC_50_	Half-maximal inhibitory concentration
LC_50_	Toxicity levels
EC_50_	Anti-settlement activity
RDC	Residual dipolar coupling
TDDFT-ECD	Time-dependent density functional theory-electronic circular dichroism
FTIR	Fourier Transform Infrared Spectroscopy
OR	Optical Rotatory
LPS	Lipopolysaccharide
sEH	Soluble epoxide hydrolase
PBMC	Peripheral blood mononuclear cells
PTP1B	Protein tyrosine phosphatase 1B
RSV	Respiratory syncytial virus
MD	Molecular dynamics
fMLF/CB	Formyl-methionyl-leucyl-phenylalanine/cytochalasin B
COX-2	Enzyme cyclooxygenase-2
PGE2	Prostaglandin E2
IgE	Immunoglobulin E
IR	Infrared
HR-APCI-MS	High-Resolution Atmospheric Pressure Chemical Ionization Mass Spectrometry
